# A Matrix-Assisted Laser Desorption/Ionization—Mass Spectrometry Assay for the Relative Quantitation of Antennary Fucosylated *N*-Glycans in Human Plasma

**DOI:** 10.3389/fchem.2020.00138

**Published:** 2020-02-28

**Authors:** Osmond D. Rebello, Simone Nicolardi, Guinevere S. M. Lageveen-Kammeijer, Jan Nouta, Richard A. Gardner, Wilma E. Mesker, Rob A. E. M. Tollenaar, Daniel I. R. Spencer, Manfred Wuhrer, David Falck

**Affiliations:** ^1^Center for Proteomics and Metabolomics, Leiden University Medical Center, Leiden, Netherlands; ^2^Ludger Ltd, Culham Science Centre, Abingdon, United Kingdom; ^3^Department of Surgery, Leiden University Medical Center, Leiden, Netherlands

**Keywords:** antennary fucose, glycomics, sialyl-Lewis x, MALDI-MS, FT-ICR-MS, CE-MS, colorectal cancer, exoglycosidase

## Abstract

Changes in the abundance of antennary fucosylated glycans in human total plasma *N*-glycome (TPNG) have been associated with several diseases ranging from diabetes to various forms of cancer. However, it is challenging to address this important part of the human glycome. Most commonly, time-consuming chromatographic separations are performed to differentially quantify core and antenna fucosylation. Obtaining sufficient resolution for larger, more complex glycans can be challenging. We introduce a matrix-assisted laser desorption/ionization—mass spectrometry (MALDI-MS) assay for the relative quantitation of antennary fucosylation in TPNG. *N*-linked glycans are released from plasma by PNGase F and further treated with a core fucosidase before performing a linkage-informative sialic acid derivatization. The core fucosylated glycans are thus depleted while the remaining antennary fucosylated glycans are quantitated. Simultaneous quantitation of α2,3-linked sialic acids and antennary fucosylation allows an estimation of the sialyl-Lewis x motif. The approach is feasible using either ultrahigh-resolution Fourier-transform ion cyclotron resonance mass spectrometry or time-of-flight mass spectrometry. The assay was used to investigate changes of antennary fucosylation as clinically relevant marker in 14 colorectal cancer patients. In accordance with a previous report, we found elevated levels of antennary fucosylation pre-surgery which decreased after tumor resection. The assay has the potential for revealing antennary fucosylation signatures in various conditions including diabetes and different types of cancer.

## Introduction

Changes in the relative abundance of either core or antennary fucosylation have been associated with certain diseases or disease states (Blomme et al., 2009; Testa et al., 2015). Here, we focus on antennary fucosylation in human total plasma *N*-glycome (TPNG) as a clinically relevant disease marker. For instance, the abundance of antennary fucosylation in TPNG has been correlated with (1) certain cancers such as hepatocellular carcinoma (Benicky et al., 2014; Zhu et al., 2014), ovarian cancer (Saldova et al., 2007) and colorectal cancer (Holst et al., 2016; de Vroome et al., 2018; Doherty et al., 2018); (2) hepatocyte nuclear factor 1 homeobox A—maturity-onset diabetes of the young (HNF1A—MODY) (Thanabalasingham et al., 2013; Juszczak et al., 2019); (3) inflammatory conditions (Brinkman-van der Linden et al., 1998; Higai et al., 2005) and (4) with attention-deficit hyperactivity disorder in children (Pivac et al., 2011). These correlations have created a need for the development of assays for quantitation of antennary fucosylation in TPNG. Quantitation of changes in core fucosylated glycans will remain relevant as well, but can better covered by existing approaches.

The quantitation of antennary fucosylation in TPNG was also used for more specialized clinical purposes such as for prognosis or for differentiating between closely related diseases. For example, a lowered incidence of antennary fucosylation on triantennary glycans was shown to discriminate HNF1A-MODY patients from Type 1 and Type 2 diabetes (Thanabalasingham et al., 2013). Current genetic tests for diagnosing HNF1A-MODY are sometimes inconclusive, and there is a demand for additional diagnostic markers (Schober et al., 2009; Thanabalasingham and Owen, 2011; Hattersley and Patel, 2017). Plasma *N*-glycome antennary fucosylation has been reported to be regulated by HNF1A making it a proxy of HNF1A expression levels and functions (Lauc et al., 2010). Hence, quantitation of antennary fucosylation in TPNG has been found to be an HNF1A-MODY disease biomarker with potential for complementing genetic tests in disease diagnosis (Lauc et al., 2010; Thanabalasingham et al., 2013). For colorectal cancer, both better diagnosis and prediction of long-term survival are urgent clinical needs. Currently, long-term survival predictions are mostly based on tumor node metastasis classification which has low success rates (Cserni, 2003; de Vroome et al., 2018). This negatively affects the decision making on therapy given to the patients. Antennary fucosylation was shown to be associated with the recovery of colorectal cancer patients after tumor resection (de Vroome et al., 2018), and thus may have the potential for long-term survival prediction.

**Figure 1:**
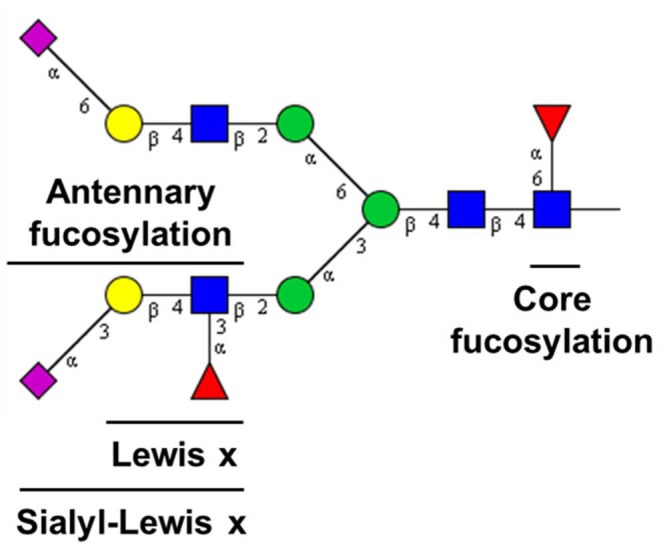
Fucose epitopes on *N*-glycans in human plasma. [Blue square: *N*-acetylglucosamine, green circle: mannose, yellow circle: galactose, red triangle: fucose, pink diamond: *N*-acetylneuraminic acid (+45° α2,6-linked; −45° α2,3-linked). For compositions see Figure 6].

TPNG is a rich and convenient source of valuable associations with diseases and disease states. It reflects the loss of systemic or cellular homeostasis which may affect the regulation of glycosylation pathways (Nairn et al., 2008; Blomme et al., 2009; Lauc et al., 2010). Enabling a bird's-eye view, TPNG analysis is highly complementary to the target analysis of specific proteins. Quantitation of low abundant antennary fucosylated glycans in human TPNG is complicated by the structural diversity of its component glycans. This diversity is a combination of monosaccharide composition variants and linkage isomers (Royle et al., 2008; Stumpo and Reinhold, 2010). Composed of diantennary, triantennary and tetraantennary *N*-glycans, TPNG also features bisecting *N*-acetylglucosamine and glycans with *N*-acetyllactosamine repeats, and complexity is added by different levels of galactosylation, sialylation and fucosylation (Stumpo and Reinhold, 2010; Vreeker et al., 2018; Lageveen-Kammeijer et al., 2019). Fucosylation can be classified as either core or antennary (Figure 1). Core fucosylation is linked by an α(1-6) glycosidic bond to the reducing end *N*-acetylglucosamine. Antennary fucosylation in TPNG mainly features fucose residues that are linked by an α(1-3) glycosidic bond (Lewis x epitope) to antennary *N*-acetylglucosamine residues (Staudacher et al., 1999).

Methods for sample preparation and analysis of human TPNG have been developed on a number of analytical platforms. This includes liquid chromatography (LC)—fluorescence detection (FLD) (Royle et al., 2008; Knezevic et al., 2011; Pivac et al., 2011; Doherty et al., 2018), MALDI-MS (Reiding et al., 2014; Bladergroen et al., 2015; Vreeker et al., 2018), and capillary gel electrophoresis—laser-induced fluorescence (Ruhaak et al., 2010; Vanderschaeghe et al., 2010). Each method has its advantages and disadvantages when it comes to measuring antennary fucosylation. Capillary gel electrophoresis—laser-induced fluorescence (Vanderschaeghe et al., 2010) and LC-FLD (Pivac et al., 2011; Doherty et al., 2018) are able to resolve some antennary fucosylated glycans in TPNG along their chromatographic dimension. However, LC-FLD often requires extensive measurement time. Nonetheless, due to its high precision and robustness, one of the most attractive techniques for routine applications is LC-FLD. MALDI-MS is gaining popularity in biomarker analysis since it features short measurement time and high molecular resolution. This, combined with linkage-informative sialic acid derivatization, is the ideal basis for an high throughput TPNG glycomic assay in a research setting (Reiding et al., 2014; Bladergroen et al., 2015; Vreeker et al., 2018). However, due to identical mass, the differentiation between core fucosylation and antennary fucosylation in monofucosylated glycans of the same composition is not achieved by MALDI-MS. Therefore, additional experiments are needed such as tandem mass spectrometry (Chen and Flynn, 2007; Wuhrer et al., 2011; Lattova et al., 2019) or exoglycosidase treatment (Royle et al., 2008). Unfortunately, relative quantitation of fucose isomers by tandem MS is hindered by the vastly different efficiencies of the fragmentation pathways (Banazadeh et al., 2019; Lattova et al., 2019). Additionally, potential fucose rearrangement influences the ratios of diagnostic fragment ion(s), further interfering with the assessment of mixtures by tandem MS (Harvey et al., 2002; Chen and Flynn, 2007; Wuhrer et al., 2011). Alternatively, endoglycosidases can be used for glycomic assays (Benicky et al., 2014; Vanderschaeghe et al., 2018). For example, a combination of Endo F2 and Endo F3 has been used to quantify antennary fucosylation on diantennary and triantennary glycans on hemopexin and complement factor H in patients suffering from liver diseases (Benicky et al., 2014). However, until now, this approach has not been demonstrated on TPNG. Due to the narrow specificity of these endoglycosidases in contrast to the vast structural diversity of TPNG, such an approach may not be suitable for TPNG.

We developed an assay for the relative quantitation of antennary fucosylation in human TPNG by combining MALDI-MS and exoglycosidase approaches. It can be viewed as complementing the existing approach for TPNG measurement by MALDI-MS. The assay was applied to 14 colorectal cancer patient samples. Consistent antennary fucosylation changes pre vs. post tumor resection were detected which revealed the assays potential for addressing biomedical research questions.

## Materials and Methods

### Reagents and Samples

Disodium hydrogen phosphate dihydrate, potassium dihydrogen phosphate, sodium chloride, 85% phosphoric acid, 30–33% (*v/v%*) ammonium hydroxide, nonidet P-40 substitute (NP-40), 1-hydroxybenzotriazole 97% (HOBt), ammonium acetate and super-DHB (9:1 mixture of 2,5-dihydroxybenzoic acid and 2-hydroxy-5-methoxybenzoic acid) were purchased from Sigma Aldrich Chemie GmbH (Steinheim, Germany). 1-Ethyl-3-[3-(dimethylamino)-propyl]carbodiimide hydrochloride (EDC) was purchased from Fluorochem (Hadfield, UK). Analytical grade ethanol, analytical grade glacial acetic acid, sodium dodecyl sulfate (SDS), trifluoroacetic acid, and potassium hydroxide were purchased from Merck KGaA (Darmstadt, Germany). HPLC-grade acetonitrile was purchased from Biosolve (Valkenswaard, The Netherlands). Girard's Reagent P (GiRP) was purchased from TCI Development Co. Ltd. (Tokyo, Japan). 5× phosphate buffered saline solution (PBS; 175 mM; pH 7.3) was prepared by dissolving 285 g of disodium hydrogen phosphate dihydrate, 23.8 g of potassium dihydrogen phosphate and 425 g of sodium chloride in 10 L deionized water. The 5× acidic PBS was prepared by adding 68 μL of 85% phosphoric acid (14.7 M) to 9.93 mL of the 5× PBS. Recombinant Peptide *N-*glycosidase F (PNGase F) was obtained from Roche Diagnostics (Mannheim, Germany). The recombinant core fucosidase, commercially known as α1-2,4,6 Fucosidase O, was purchased from New England BioLabs (MA, USA). However, activity on the α1,4-linkage is reported as very low. Under the employed conditions, the enzyme did not noticeably act on antennary fucoses present in TPNG (see section *Result and Discussion*). Peptide Calibration Standard II was purchased from Bruker Daltonic (Bremen, Germany).

Human plasma standard (Visucon-F frozen normal control plasma, pooled from a minimum of 20 human donors, citrated and buffered in 0.02 M 4-(2-hydroxyethyl)-1-piperazineethanesulfonic acid) was purchased from Affinity Biologicals (Ancaster, Ontario, Canada).

The pre-operative vs. 45 days post-operative pairs of 14 colorectal cancer patient samples were collected as part of a biobank as was previously described (de Vroome et al., 2018). These serum samples were collected between October 2002 and March 2013 by the Leiden University Medical Center Surgical Oncology Biobank. This study was approved by the Medical Ethics Committee of the Leiden University Medical Center and was performed in accordance with the Code of Conduct of the Federation of Medical Scientific Societies in the Netherlands (http://www.federa.org/).

### *N*-Glycan Release

PNGase F release of human TPNG was performed similarly as previously described (Vreeker et al., 2018). Briefly, 4 μL of plasma was added to 8 μL of 2% SDS in a polypropylene 96 well V-bottom plate (V-96 microwell, NUNC, Roskilde, Denmark). The plate was sealed (adhesive plate seals, Thermo Scientific, UK) and mildly shaken on a plate shaker for 5 min, before incubating at 60°C for 10 min. Additionally, 8 μL of the PNGase F releasing mixture (4 μL of 4% NP-40 solution, 4 μL of 5× acidic PBS and 0.4 μL of PNGase F) was then added to the plasma samples. The plate was sealed and mildly shaken on a plate shaker for 5 min before incubating overnight (15–18 h) at 37°C.

### Depletion of Core Fucosylation

The overnight incubated plasma release mixture (5 μL) was diluted with 45 μL of 1× acidic PBS solution in a 96 well V-bottom plate (V-96 microwell, Grenier Bio-one, Germany). The plate was mildly shaken on a plate shaker for 5 min, before transferring 1–2 μL of core fucosidase mixture (0.65 μL deionized water, 0.75 μL of 4% NP-40 solution, 0.4 μL of 5× acidic PBS and 0.2 μL/0.4 Units of core fucosidase) in a 96 well V-bottom plate. After sealing of the plate, the samples were incubated overnight (15–18 h) at 37°C in an enclosed, humidified chamber to prevent evaporation.

### Linkage Specific Sialic Acid Derivatization

Sialic acid derivatization was performed as previously described (Lageveen-Kammeijer et al., 2019), but with minor alterations. With this approach, the carboxylic acid groups of α(2,6)-linked sialic acids are ethyl esterified while the α(2,3)-linked sialic acids are amidated. Briefly, 60 μL of ethyl esterification reagent (solution of 0.25 M EDC and 0.25 M HOBt in ethanol) was added to the core defucosylated samples. The plate was sealed and incubated at 37°C for 30 min. Twelve microliters of 30–33% (*v/v%*) NH_4_OH solution were added to the wells and the sealed plate incubated for another 30 min at 37°C. Seventy-two microliters of acetonitrile was added to the wells, after which cotton hydrophilic interaction liquid chromatography (HILIC)—solid-phase extraction (SPE) microtip purification was performed immediately.

### Cotton Hydrophilic Interaction Liquid Chromatography—Solid-Phase Extraction Microtip Purification

This purification step was performed as previously described (Selman et al., 2011) with minor modifications. The cotton HILIC-SPE microtips were prepared by inserting a cotton strand of length 3–4 mm into a 20 μL capacity microtip (Mettier-Toledo, Switzerland) and pushing it into place with a stream of pressurized air/nitrogen. Conditioning was performed by pipetting 5 times 20 μL of water, followed by an equilibration step of pipetting 3 times 20 μL of 85% acetonitrile. The derivatized samples were loaded onto the HILIC-SPE by pipetting 20 μL of it 20 times. Washing was performed with 3 times 20 μL of 85% acetonitrile containing 1% trifluoroacetic acid and 3 times 20 μL of 85% acetonitrile, consecutively. The glycans were eluted from the HILIC-SPE by repeatedly, 10 times, pipetting 4 μL of deionized water in a 96 well V-bottom plate (V-96 microwell, Grenier Bio-one, Germany). All steps were performed with a 12 channel multi-channel pipette.

Purified glycan samples (1 μL) were spotted on an MTP anchor chip 600/384 TF MALDI target plate (Bruker Daltonics, Bremen, Germany), followed by the addition of 1 μL of the MALDI matrix solution (50% acetonitrile solution of 2.5 mg/mL super-DHB and 0.1 mM sodium hydroxide). The solutions were mixed on the plate with a pipette. The spotted samples were allowed to air dry before performing MALDI- time-of-flight -MS (MALDI-TOF-MS) or MALDI- Fourier-transform ion cyclotron resonance -MS (MALDI-FT-ICR-MS) measurements.

### MALDI-TOF-MS Analysis

The analysis was performed on an UltrafleXtreme Mass spectrometer in reflectron positive ion mode (Bruker Daltonics, Bremen, Germany) which was operated by FlexControl version 3.4 (Build 135). A Bruker Smartbeam-II laser was used for ionization at an irradiation frequency of 1 kHz using the “small” predefined laser shot pattern. Each sample spot was irradiated by 20,000 shots with 200 shots at each laser raster. Irradiation was performed randomly over the complete sample spot. Spectra were recorded within an *m/z* range of 900–5,000. Samples were measured in an automated manner using the AutoXecute function of FlexControl. Before each measurement, the instrument was calibrated with a peptide standard mix (Peptide Calibration Standard II, Bruker Daltonics).

### MALDI-FT-ICR-MS Analysis

The analysis was performed in positive ion mode on a Bruker 15T solariX XR FT-ICR mass spectrometer equipped with a CombiSource and a ParaCell (Bruker Daltonics). The system was operated by ftmsControl version 2.2.0 (Build 150). A Bruker Smartbeam- II laser was used for ionization at an irradiation frequency of 500 Hz using the “medium” predefined laser shot pattern. Each sample spot was irradiated with a raster of 200 laser shots. Ten such scans were performed randomly over the complete sample spot. Spectra were acquired within an *m/z*-range 1,011–5,000 with 1 million data points (transient time 2.3069 s). Samples were measured in an automated manner using the AutoXecute function of ftmsControl.

### Processing of MALDI-MS Data

The MALDI-TOF-MS spectra were internally calibrated using FlexAnalysis version 3.4 (Build 76; Bruker Daltonic). The glycan calibrants used were [H3N4 + Na]^+^ of *m/z* 1,339.476, [H5N4 + Na]^+^ of *m/z* 1,663.581, [H5N4E1 + Na]^+^ of *m/z* 1,982.708, [H5N4E2 + Na]^+^ of *m/z* 2,301.835, [H5N5E2 + Na]^+^ of *m/z* 2,504.914, [H6N5E2Am1 + Na]^+^ of *m/z* 2,957.078 and [H7N6E2Am2 + Na]^+^ of *m/z* 3,612.322. The.xy files generated by Flexanalysis were further processed with Massy Tools (Jansen et al., 2015). A second round of internal spectral calibration was performed with at least four calibrants in the low *m/z* range and at least three calibrants in the medium *m/z* range, having to pass signal to noise ratio (S/N) at least above 15. The *m/z* window for calibration, peak detection, and spectral data integration was taken at ± 0.45 Th. The background detection windows were set to 20 Th.

The MALDI-FT-ICR-MS spectra were acquired in serial mode. Thus, a single combined file was generated. This file was split into individual compound spectra and transformed into.xy files using DataAnalysis version 5 (Bruker Daltonics). Calibration of these spectra was performed in Massy Tools, for which at least four calibrants in the low *m/z* range and at least three calibrants in the medium *m/z* range, have to pass S/N at least above 50. The *m/z* window for calibration was taken at ± 0.10 Th. Peak detection and spectral data integration was performed using an extraction window depending on the defined glycans in the analyte list. This was done so as to exclude interferences in the spectra. The background detection windows were set at the value of 20 Th.

The calibrant list and analyte list for MALDI-TOF-MS spectra and MALDI-FT-ICR-MS spectra are shown in Supplementary Tables S1, S2, respectively. The table of all identified and quantitated glycans by MALDI-FT-ICR-MS and MALDI-TOF-MS, respectively, are shown in Supplementary Table S3. At least 85% of the theoretical isotopic distribution of each glycan analyte was integrated. The Massy tools output was used further for analyte curation, which was based on multiple criteria. These were S/N ≥ 9, isotopic pattern quality ≤ 0.25 and mass accuracy between ± 20 ppm (MALDI-TOF-MS) or ± 10 ppm (MALDI-FT-ICR-MS). Spectra for which the number of glycan analytes passing these criteria were <50% of the total number of defined glycans in the analyte list, were not considered for further processing as these spectra were deemed of low quality. The absolute area of the curated glycans was corrected to 100% of their respective isotopic distribution, before using them in total area normalization.

Derived traits, focusing on features rather than individual glycans, were calculated from the relative areas of the glycan compositions using an in-house prepared R script (Supplementary Table S4). Statistical significance of the differences between pre-operative vs. post-operative colorectal cancer patient samples was assessed by a Wilcoxon matched-pairs signed-rank test with α = 0.05. Multiple-testing correction was performed using a false discovery rate of 1% calculated with the Benjamini and Hochberg method. These statistical tests were performed in GraphPad Prism version 8.0.1. The box and whisker plots used for representing the features were made by an in-house prepared R script.

### Method Optimization and Assay Performance

All optimization experiments of the assay were measured on the MALDI-TOF-MS (Supplementary Material “*Assay optimization*”). Data processing for these mentioned optimization experiments was done using the mass list of the negative control (fucosidase untreated TPNG) (Supplementary Table S2). Quantitation of residual core fucosylated glycans allowed the assessment of the completeness of the core defucosylation. Importantly, all identified antennary fucosylated glycans were also included in the mass list of the negative control (fucosidase untreated TPNG).

The intermediate precision of the antennary fucose assay for combined sample preparation and measurements on the MALDI-TOF-MS or MALDI-FT-ICR-MS were performed in three independent experiments on three different days within a period of 5 days. For each of these experiments, the assay was performed with nine replicates of glycan releases from a human plasma pool (Visucon-F, Affinity Biologicals). Data processing was performed as mentioned in the subsection “*Processing of MALDI-TOF-MS/MALDI-FT-ICR-MS data*”.

### Capillary Electrophoresis—Electrospray Ionization—Mass Spectrometry

Identification of core fucosylated glycans, antennary fucosylated glycans, and composition containing both isomers was done by comparing the MALDI-FT-ICR-MS spectra from the assay (core fucosidase treated) to the negative control (fucosidase untreated). The identified antennary fucosylated glycans and mixed fucosylated isomeric glycans were structurally confirmed by targeted collision induced dissociation (CID) fragmentation on a capillary electrophoresis—electrospray ionization–tandem MS (CE-ESI-MS/MS) platform. The samples were performed in 12 replicates of which nine were used for MALDI-FT-ICR-MS measurements while the remaining three were used for CE-ESI-MS/MS measurements (*n* = 3).

For the CE-ESI-MS/MS measurement, the replicates from the assay and the negative controls were, respectively, pooled together and dried down in a vacuumed centrifuge (Salm en Kipp, Breukelen, Netherlands) at 50°C. They were used for permanent cationic labeling with GiRP as previously described (Lageveen-Kammeijer et al., 2019) but with certain alterations. Briefly, 10 μL of GiRP labeling solution (7.5 mg GiRP dissolved in a solution of 720 μL ethanol and 80 μL glacial acetic acid) was added to the dried samples. The plate was sealed and mildly shaken on a plate shaker for 5 min before incubating at 60°C for 1 h. After incubation, the samples were dried down in a vacuumed centrifuge at 50°C and then re-suspended in 5 μL of deionized water. In total, 3.6 μL of the GiRP labeled glycans were mixed with 2.4 μL of 250 mM ammonium acetate solution as leading electrolytes (250 mM ammonium acetate solution adjusted to pH 4 with glacial acetic acid) before being transferred into a vial (nanoVial, Sciex, Framingham, USA).

All CE-ESI-MS/MS analyses were performed on a static coated neutral capillary cartridge (Neutral OptiMS cartridge, Sciex), fitted into a CESI 8000 system (Sciex). The CE system was coupled with an Impact HD UHR-QTOF-MS system (Bruker Daltonics). When the capillary was not in use, a continuous flow of water at 10 psi was applied to the separation line. Prior to usage, the separation line and reverse line were filled with the background electrolyte containing 10% acetic acid (sonicated in a water bath for 10 min). Prior to sample injection, the separation line was flushed with 0.1 M HCl at a pressure of 100 psi for 5 min. This was followed by flushing the reverse line with background electrolyte at 75 psi for 3 min. The separation line was filled with background electrolyte by applying a pressure of 100 psi for 10 min. The sample was injected into the separation line of the capillary from the nanovials via a hydrodynamic injection of 12.5 psi for 24 s (about 5% of the capillary volume). The tip of the separation line was washed by momentary dipping it into a vial of background electrolyte, followed by injecting a background electrolyte plug with a pressure of 2.5 psi for 15 s. A 20 kV voltage of normal polarity (cathode toward the end of the capillary) was applied on the capillary for 30 min. During this step, a continuous flow of 2 psi was applied only on the reverse line. After 30 min, a flow of background electrolyte is applied to both the separation line and reverse line by applying a pressure of 2 psi on both lines for 40 min. Finally, the voltage on the capillary was ramped down over 5 min to 1 kV before termination of the run. The capillary was maintained at 30°C throughout the analysis.

For the MS analysis, a dopant enriched nitrogen gas (acetonitrile as dopant) at 0.2 bar was used for nebulization at the ESI captive sprayer. The drying gas of nitrogen at 150°C was introduced at the source at 1.2 L/min and the internal capillary of the MS was maintained at 1,200 V. A targeted CID fragmentation was performed on 20 glycan analytes of interested. This list was divided into two inclusion lists on the software otof control version 3.4 (Build 14; Bruker Daltonics) which was used for operating the MS and MS/MS analysis. Hence each sample was measured twice in-order to fragment all the glycan analytes of interest. The MS/MS fragmentation spectra were collected at a rate of 1 Hz within an *m/z*-range of 150–2,000 and at an absolute intensity threshold at 2,274 on the *m/z* values of interest. The targeted precursor ions were isolated with a width of 8–15 Th depending on the *m/z* values. The collision energies of the CID cell was set in an *m/z* dependent manner, ranging from 35 eV for singly charged precursor ions of *m/z* 500 to 70 eV for singly charged precursor ions at *m/z* 2,000. Data analysis was done using DataAnalysis version 5 (Bruker Daltonics).

### Hydrophilic Interaction Liquid Chromatography Analysis

PNGase F released *N*-glycans from 12 replicates of human plasma samples (Visucon-F) were used for procainamide labeling and analysis on a HILIC-FLD-MS^n^ platform as previously described (Kozak et al., 2015). Following PNGase F treatment, 8 μL of each sample was dried down and the released *N*-glycans converted to aldoses with 0.1% formic acid, filtered through a protein binding plate (LC-PBM-96, Ludger, Oxford, UK), washed twice with 100 μL of water and dried. *N*-glycans were labeled by reductive amination in 10 μL of water and 10 μL procainamide labeling solution (LT-KPROC-24 containing NaCNBH_3_, Ludger) and incubated at 65°C for 1 h. A HILIC-type clean-up plate (LC-PROC-96, Ludger) was used to remove unreacted procainamide dye. Procainamide labeled *N*-glycans were eluted in 300 μL of water. The samples were dried and resuspended in water (50 μL) for further analysis.

Procainamide-labeled samples were analyzed by HILIC-FLD-MS^n^. 12.5 μL of each sample was injected into an ACQUITY BEH Glycan column (1.7 μm, 2.1 × 150 mm) at 40°C on a Dionex Ultimate 3000 UHPLC instrument with a fluorescence detector (ex = 310 nm and em = 370 nm attached to an Amazon Speed ETD (Bruker Daltonics). The running conditions used were as follows: solvent A was 50 mM ammonium formate (pH 4.4) (LS-NBUFFX40, Ludger), and solvent B was acetonitrile.

Gradient conditions were as follows: 0–53.5 min, 76–51% B, 0.4 mL/min; 53.5–55.5 min, 51–0% B, 0.4–0.2 mL/min; 55.5–57.5 min, 0% B at a flow rate of 0.2 mL/min; 57.5–59.5 min, 0–76% B, 0.2 mL/min; 59.5–65.5 min, 76% B, 0.2 mL/min; 65.5–66.5 min, 76% B, 0.2–0.4 mL/min; 66.5–70.0 min, 76% B, 0.4 mL/min. The Amazon Speed settings used were as follows: source temperature, 250°C; gas flow, 10 L/min; capillary voltage, 4,500 V; ICC target, 200,000; Max. accu. time (Maximum Accumulation Time), 50.00 ms; rolling average, 2; number of precursor ions selected, 3; release after 0.2 min; positive ion mode; scan mode, enhanced resolution; mass range scanned, 400–1,500; target mass, 900.

## Results and Discussions

**Figure 2:**
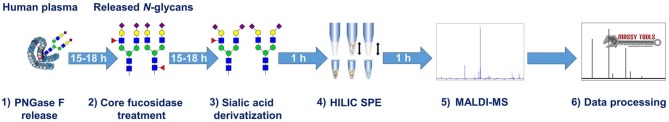
Workflow of the antennary fucose assay for human plasma.

The workflow for the antennary fucose assay is shown in Figure 2. Released *N*-glycans from human plasma were treated with an α1,6-linkage-selective fucosidase (core fucosidase) to deplete core fucosylation. Antennary fucosylation remained and was relatively quantified by MALDI-FT-ICR-MS or MALDI-TOF-MS. Information on sialic linkage is obtained in parallel, potentially improving the estimation of sialyl-Lewis x abundance. An overview of all analytical methods used during the development of the assay is shown in Supplementary Figure S1.

**Figure 3:**
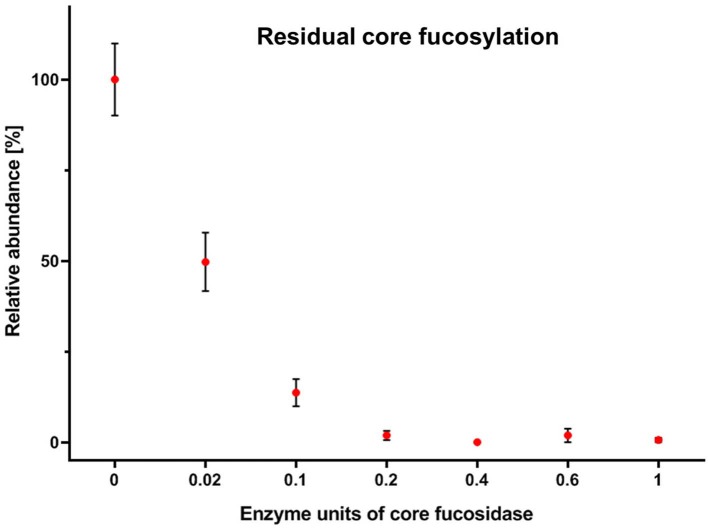
Residual core fucosylation on TPNG after incubation with varying amounts of core fucosidase. The amounts of core fucosylation on complex *N*-glycans were calculated and were normalized to their lowest and highest mean values. Error bars show the standard deviation of the mean of three replicates.

Human TPNG was 28.3% ± 0.5% fucosylated. Most of this is core fucosylation (Supplementary Table S5). Hence, it was important to optimize the assay with a focus on a robust and complete depletion of core fucosylation of TPNG. This aids a robust relative quantitation of the remaining antennary fucosylated glycans by MALDI-MS. The efficient use of costly reagents, specifically the core fucosidase, was also addressed. During the optimization steps, completeness of core defucosylation was judged by the inability to quantify by MALDI-MS (signal to noise ratio (S/N) <9) some of the most abundant core fucosylated glycans namely [H3N4F1 + Na]^+^ of *m/z* 1,485.53 and [H4N4F1 + Na]^+^ of *m/z* 1,647.59 (Supplementary Figure S4). Details pertaining to assay optimization are described in the Supplementary Material section “*Assay optimization*.” For these optimized assay conditions, a complete core defucosylation of TPNG could already be achieved with 0.2 units of core fucosidase (Figure 3). However, to facilitate robustness of the core defucosylation a 2-fold greater amount (0.4 units) was chosen for further experiments. Equally important is the preservation of antennary fucosylation. A fucosidase specific, or at least highly selective, for the α1,6-linkage over other linkages significant in TPNG (mainly α1,3-linkage) is therefore essential. The MS/MS spectra provided in the Supplementary Material demonstrate that core fucoses are removed while antennary fucoses remain (see also following sub-section) with the chosen enzyme. The integrity of the antennary fucosylation under increased enzyme concentrations (Figure 3) and incubation times (data not shown) further supports the α1,6-linkage selectivity. An absence of the oxonium ion of *m/z* 658.26 [galactose—*N*-acetylglucosamine(fucose)_2_+H]^+^ excluded a significant presence of Lewis y or Lewis b structures.

### Identification of Antennary Fucosylated Glycans in Total Plasma *N*-Glycome

**Figure 4:**
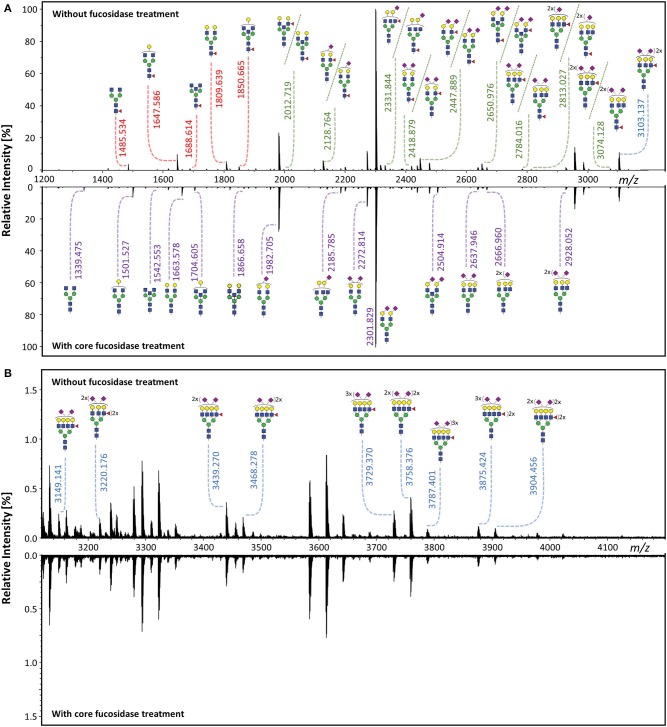
Identification of core fucosylated glycans and antennary fucosylated glycans by exoglycosidase and MALDI-FT-ICR-MS. The TPNG profile without core fucosidase treatment was compared to the profile after treatment, within **(A)** the *m/z* range of 1,200–3,200 and **(B)** the *m/z* range of 3,120–4,200. Core fucosylated glycans [red *m/z* values] are converted to their corresponding afucosylated glycans [purple *m/z* values], upon core fucosidase treatment. Only the antennary fucosylated glycans [blue *m/z* values] and the antennary fucose isomers of the mixed fucose isomeric (core or antennary fucosylation) glycans [green *m/z* values] remain after core fucosidase treatment. All *m/z* values of annotated glycans belong to [M + Na]^+^ ions. The description of the glycan cartoons are shown in Figure 1.

**Figure 5:**
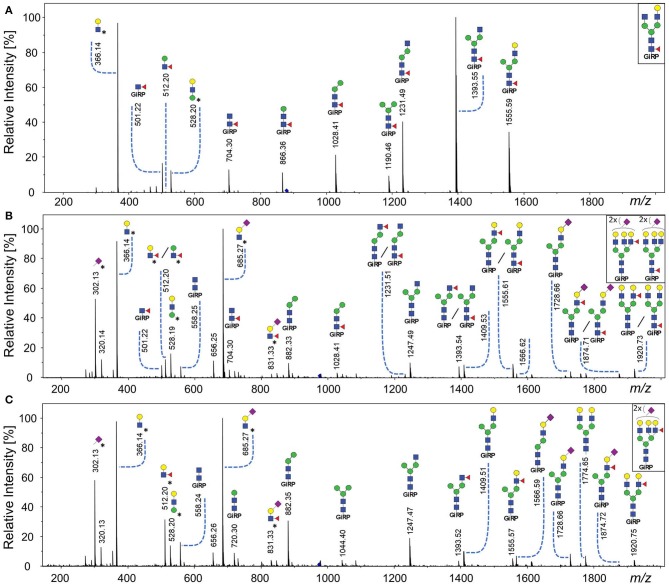
CE-ESI-MS/MS spectra of sialic acid derivatized and GiRP labeled TPNG for the confirmation of antennary, mixed isomeric (core or antennary fucosylation) and core fucosylated glycans. **(A)** The [M+H]^2+^ ion of H4N4F1 (*m/z* 879.838) is fragmented for fucosidase untreated TPNG. Its fragment ions are indicative of core fucosylation. **(B)** The [M+2H]^3+^ ion of H6N5F1E2 (*m/z* 975.709) [second isotope] is fragmented for fucosidase untreated TPNG. Its fragment ions are indicative of both core fucosylated and antennary fucosylated isomers. **(C)** The ion [M+2H]^3+^ of H6N5F1E2 (*m/z* 975.709) [second isotope] is again fragmented, but for core fucosidase treated TPNG. Its fragmentation pattern is indicative of only antennary fucosylation. The descriptions of the glycan cartoons are shown in Figure 1. GiRP represents Girard's reagent P label; * represents an oxonium ion.

Core fucosylated glycans and antennary fucosylated glycans in TPNG were identified by comparing the TPNG profile with and without core fucosidase treatment (Figure 4). A more detailed view can be found in Supplementary Figures S2, S3 for MALDI-FT-ICR-MS and MALDI-TOF-MS spectra, respectively. Interestingly, some monofucosylated glycan compositions in TPNG showed a mixture of both core and antennary fucosylated isomers (Supplementary Figure S4). CID spectra, obtained on a CE-ESI-MS/MS platform, provided an orthogonal layer of evidence (Figure 5). CID spectra for all identified antennary fucosylated glycans are shown in Supplementary Figures S5–S24. Antennary fucosylation was identified by the formation of the diagnostic B-ion of *m/z* 512.198 assigned as [galactose-*N*-acetylglucosamine(fucose)+H]^+^ (Wuhrer et al., 2011). Core fucosylation was identified by the formation of the Y-ion of *m/z* 501.219 assigned as [*N*-acetylglucosamine(fucose)-GiRP]^+^. Core fucosylation also generates, to a lesser extent, the B-ion of *m/z* 512.198 assigned as [mannose-*N*-acetylglucosamine(fucose)+H]^+^ caused by fucose rearrangement (Harvey et al., 2002; Chen and Flynn, 2007; Wuhrer et al., 2011; Lettow et al., 2019). This complicates the assessment of mixtures, especially relative quantitation by MS/MS. Although, fucose rearrangement also limits the sensitivity of antennary fucose identification in the presence of core fucose isomers, significant contributions of antennary fucose are still readily identified (Figure 5).

Notably, no core fucosylation remained after core fucosidase treatment. This was assessed by the absence of the core fucosylated glycans H3N4F1and H4N4F1 (S/N < 9) (Supplementary Figure S25). As expected the corresponding afucosylated glycoforms increased in abundance (Figure 4). The remaining fucosylated glycans are suggested to be solely antennary fucosylated.

As similar relative abundances were maintained after core fucosidase treatment, the species, H6N5F2Am2E1,H6N5F1Am1E2,H7N6F1Am1E1,H7N6F1Am2E1,H7N6F1Am1E2,H7N6F1Am3E1,H7N6F2Am3E1,H7N6F1Am2E2,H7N6F2Am2E2,and H7N6F1Am1E3,are assigned as antennary fucosylated in human TPNG. This was confirmed by MS/MS (Supplementary Figures S14–S23). Interestingly, we were able to prove that the difucosylated glycans, H6N5F2Am2E1, H7N6F2Am3E1, and H7N6F2Am2E2, contained only antennary fucosylation and no core fucosylation. Previously, these glycans have often been assigned as containing one core and one antennary fucose (Vreeker et al., 2018). In fact, we were unable to identify any multifucosylated glycan compositions in TPNG having both core and antennary fucose residues on the same glycan. However, at low abundances (<0.5%), two such mixed, multifucosylated glycans, H6N5F2Am2E1 and H6N5F2Am1E2, have been convincingly demonstrated (Lageveen-Kammeijer et al., 2019). On haptoglobin, multifucosylated glycans having both core and antennary residues were shown to have clinical relevance in subtyping hepatocellular carcinoma patients (Zhu et al., 2014). Thus, it may be very important to differentiate between glycans with multiple antennary fucoses and glycans containing both a core and an antennary fucose. The former may be directly assessed with our assay, the latter indirectly, either via an increase of the resulting monofucosylated species or by comparison to the negative control (fucosidase untreated).

A range of monofucosylated compositions were found to be a mixture of core fucosylated and antennary fucosylated isomers, including for the following glycan compositions: H5N5F1, H5N4F1E1, H5N5F1E1, H5N4F1Am1E1, H5N4F1E2, H5N5F1E2, H6N5F1Am1E1, H6N5F1E2, and H6N5F1Am2E1. Their relative abundances were significantly lowered, but signals did not disappear after core fucosidase treatment (Supplementary Figure S4 and Supplementary Table S4). These findings were further supported by their MS/MS spectra (Supplementary Figures S5–S13). As a representative example, the MS/MS fragmentation of the monofucosylated composition H6N5F1E2 is shown without and with core defucosylation in Figures 5B,C, respectively. Fragmentation of this glycan from untreated human TPNG resulted in the formation of similar abundances of both a Y-ion of *m/z* 501.219 and a B-ion of *m/z* 512.198. This is typical of a mixture of core and antennary fucosylation (Figure 5B). Expectedly, the fragmentation of this glycan after core defucosylation, results in the formation of only the B-ion of *m/z* 512.198 which is indicative of only antennary fucosylation (Figure 5C). Thus, H6N5F1E2 in human TPNG is comprised of a mixture of core fucose isomers and antennary fucose isomers. Such monofucosylated glycan compositions which include mixtures of core fucose and antennary fucose isomers contribute to a total abundance of 12.1% ± 1.0% in human TPNG. Our assay determined 3.1% ± 0.3% of them to be antennary fucosylated (Supplementary Table S5).

A B-ion of *m/z* 831.325 was observed in several CID spectra which was tentatively assigned as [*N*-acetylneuraminic acid(amidated)-*N*-acetylglucosamine(fucose)+H]^+^. This is an unconventional motif, because antenna fucosylation is generally observed on α2-3 sialylated antennae. The ion may be caused by fucose rearrangement between the antennae (Wuhrer et al., 2011). For the composition H6N5F1E2 (Figure 5C), the higher abundance of the B-ion of *m/z* 512.198 compared to *m/z* 831.325, indeed indicates a Lewis x structure to be more likely. Additionally, for the compositions with only α2-6 sialylated antennae, H5N4F1E2 and H5N5F1E2, we did not observe a B-ion of *m/z* 831.325 but rather the Y-ion of *m/z* 501.219 (Supplementary Figures S9B, S10B). Thus, these compositions are partly explained by incomplete core defucosylation. Their antennary fucosylated portion, suggested by the significant abundance of the Y-ion of *m/z* 512.198, could be due to side-products of the esterification of α(2-3) linked sialic acids (Toyoda et al., 2008; Pongracz et al., 2019; Suzuki et al., 2019).

After core fucosidase treatment, H6N5F2Am2E1 and H7N6F1Am1E2 showed a lower abundance (Supplementary Table S6). However, from the MS/MS spectra without fucosidase treatment, core fucosylation could not be confirmed on these glycans due to the lack of the Y-ion of *m/z* 501.219 (Supplementary Figures S16A, S18A). H6N5F2Am2E1 and H7N6F2Am2E1 both contribute to <0.25% to TPNG, and the ratio of their abundances for *core fucosidase treated / untreated* is 0.83 and 0.88, respectively (Supplementary Table S5). Thus, if their core fucosylated isomers are present in TPNG, they might be too low abundant ( ≤ 0.03%) to be identified in MS/MS spectra of our CE-ESI-MS/MS platform. Furthermore, a complete depletion of core fucosylation for H5N5F1, H5N4F1E1, H5N5F1E1, H5N4F1E2, and H5N5F1E2 was not achieved as the Y-ion of *m/z* 501.219 was still observed in their MS/MS spectra, although the B-ion of *m/z* 512.198 is equally, if not more abundant (Supplementary Figures S5–S7, S9, S10). However, most of the core fucosylation was removed (Supplementary Figure S4). Due to the relatively low abundance of the affected glycans, this small overestimation is not likely to have a significant impact on the measurements. A pessimistic estimate is an 0.1% bias in relative quantitation of total antennary fucosylation (<3% for affected glycans, such as H5N4F1E1).

### Assay Performance

**Figure 6:**
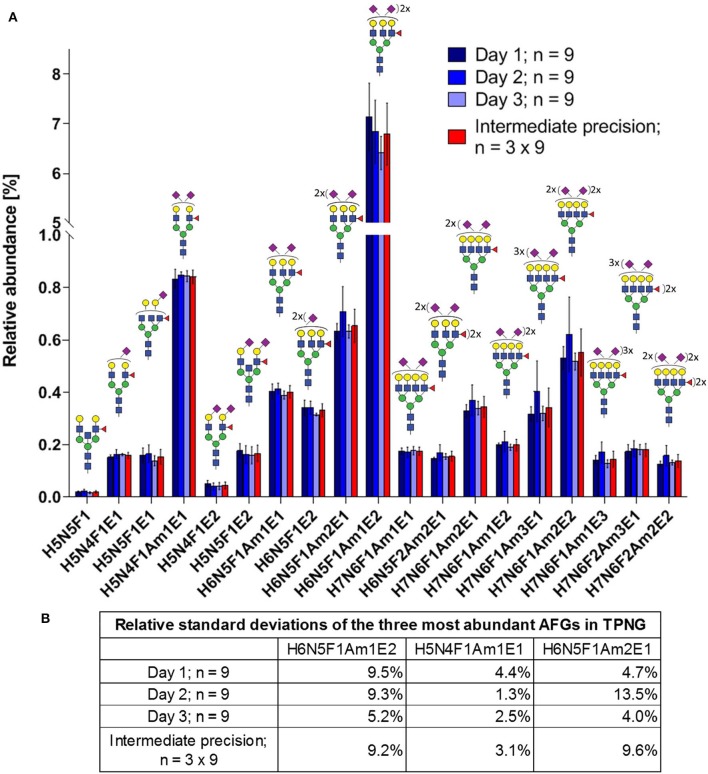
Intermediate precision of sample preparation for assay and MALDI-FT-ICR-MS measurements of antennary fucosylated glycans in human TPNG. **(A)** The mean relative abundances of the 19 quantified antennary fucosylated glycans are shown with the error bars representing standard deviation (*n* = 9). **(B)** The relative standard deviations of the three most abundant antennary fucosylated glycans are shown. The description of the glycan cartoons are as described in Figure 1. [H = hexose, N = *N*-acetylhexosamine, F = deoxyhexose (fucose), Am = amidated *N*-acetylneuraminic acid (α2,3-linked), E = ethyl esterified *N*-acetylneuraminic acid (α2,6-linked)].

Intermediate precision was assessed by three independent experiments on different days each with nine replicates of glycan releases from a human plasma pool measured by MALDI-FT-ICR-MS. Seventy glycans were quantified (Supplementary Table S7, Supplementary Figure S26), which included 19 antennary fucosylated glycans (Figure 6). These antennary fucosylated glycans make up 11.8% ± 0.9% of total abundance in human TPNG, with the three most abundant antennary fucosylated glycans, H6N5F1Am1E2, H5N4F1Am1E1, and H6N5F1Am2E1, contributing 6.80% ± 0.63%, 0.84% ± 0.03% and 0.66% ± 0.06%, respectively (Figure 6B). The abundance of H6N5F1Am1E2 is consistent with previous quantitation using MALDI-FT-ICR-MS (Vreeker et al., 2018). The median of intermediate precisions for the 19 quantitated antennary fucosylated glycans is 12.4% (9.1–18.5% interquartile range; Supplementary Figure S27), which is in-line with the ca. 10% previously described for MALDI-FT-ICR-MS analysis of all TPNG glycans (Vreeker et al., 2018). Notably, neither the focus on low abundant antennary fucosylated glycans (<1%, except H6N5F1Am1E2) nor the additional processing steps resulted in a marked loss of precision.

Previous research quantified 21 antennary fucosylated glycans in human TPNG using a MALDI-FT-ICR-MS platform (Vreeker et al., 2018). These antennary fucosylated glycans are consistent with our findings. However, we were also able to identify a mixture of both core fucose isomers and antennary fucose isomers for nine of these monofucosylated glycan compositions (Supplementary Figure S4). For example, we have identified H6N5F1E2 and H5N4F1Am1E1 as being a mixture of core fucose isomers and antennary fucose isomers. Previously, these glycans were assumed to be antennary fucosylated (H6N5F1E2) and core fucosylated (H5N4F1Am1E1), respectively. The specific measurement of antennary fucosylated glycans using our assay may increase the accuracy of the relative quantitation of antennary fucose.

To demonstrate the accuracy of the quantitation of antennary fucosylated glycans, procainamide labeled human TPNG was analyzed on a HILIC-FLD-MS^n^ platform (Kozak et al., 2015). This analytical platform was chosen for its accuracy and precision of measurements. Antennary fucosylated glycan peaks were identified from their CID spectra while relative quantitation was performed from the FLD chromatograms. By using the FLD chromatogram instead of the MS spectra for quantification, we overcome the ionization bias resulting from charge differences conferred by underivatized sialic acids (which applies to most antennary fucosylated glycans), as compared to the neutral glycans (Wheeler et al., 2009). As the HILIC-FLD-MS^n^ platform did not identify antennary fucosylated tetraantennary glycans, we based the comparison to our MALDI-MS method on antennary fucosylation of triantennary glycans only. This includes nearly 70% of the total abundance of antennary fucosylated glycans in TPNG (Supplementary Table S7). With 32.4 ± 1.1% result from our MALDI-MS method were highly comparable results to the 34.7 ± 1.3% quantified with the HILIC-FLD-MS^n^ reference method. Thus, our assay is capable of accurately quantifying antennary fucosylation in TPNG.

MALDI-FT-ICR-MS is not widely available. Therefore, we also demonstrated that the assay can be measured with a somewhat more widespread MALDI-TOF-MS instrument (Supplementary Figure S28). In total, 58 glycans could be relatively quantitated of which 15 were antennary fucosylated glycans with a total abundance of 7.3% ± 0.7% using MALDI-TOF-MS (Supplementary Table S8). In contrast, 19 antennary fucosylation glycans out of 70 glycans in total were quantified with a total abundance of 11.8% ± 0.9% using MALDI-FT-ICR-MS (Supplementary Table S7). Thus, fewer antennary fucosylated glycans were quantified with MALDI-TOF-MS due to the expectedly lower sensitivity of the instrument. Furthermore, the differences in quantified antennary fucosylation between the instruments can be accounted for by the different efficiencies of ionization and ion transfer to the detector over the *m/z* range; the MALDI-FT-ICR-MS having been more efficiently tuned for good sensitivity in the high mass range. Consistent with the overall trend, the three most abundant antennary fucosylated glycans also show lower (or equal) values in the MALDI-TOF-MS, H6N5F1Am1E2, H5N4F1Am1E1, and H6N5F1Am2E1, contributing to 3.9% ± 0.4%, 0.81% ± 0.07% and 0.41% ± 0.05% of the total abundance, respectively (Supplementary Figure S28B). The abundance of H6N5F1Am1E2 is consistent with previous MALDI-TOF-MS analysis of TPNG (Reiding et al., 2014; Bladergroen et al., 2015). The median of intermediate precisions for the 15 quantitated antennary fucosylated glycans is 12.5% (10.6–13.6% interquartile range; Supplementary Figure S29), which is virtually identical to our MALDI-FT-ICR-MS measurements of the 19 antennary fucosylated glycans. The intermediate precision of all *N*-glycans quantitated by MALDI-TOF-MS is shown in Supplementary Figure S30. Thus, the assay measured on either instrument can be used for detailed quantitation of antennary fucosylation in human TPNG.

### Quantitation of Antennary Fucosylation in Colorectal Cancer Patient Samples

To demonstrate the applicability of the developed antennary fucose assay and especially its ability to reveal clinically relevant markers of antennary fucosylation, total serum *N*-glycome (TSNG) samples were analyzed from colorectal cancer patients pre and post tumor resection. This is of specific interest, as colorectal cancer has been associated with an increase in antennary fucosylation and a decrease in core fucosylation, next to an increase in *N*-glycan antennarity and sialylation (de Vroome et al., 2018; Doherty et al., 2018). Previously, TSNG has been analyzed by MALDI-TOF-MS on sample pairs (pre-operative vs. post-operative) of 61 colorectal cancer patients from the same cohort (de Vroome et al., 2018). The derived traits that were a proxy for antennary fucosylation on *N*-glycans are especially relevant to our study. Multifucosylation on triantennary glycans and α2,3-sialylation per antenna in fucosylated triantennary glycans were significantly lowered in the post-operative patient samples as compared to pre-operative samples (de Vroome et al., 2018). The latter derived trait was used as a proxy for sialyl-Lewis x epitopes in TSNG. These changes were thought to be associated with the recovery of the patients since values were closer to healthy controls in the post-operative than in the pre-operative samples.

**Figure 7:**
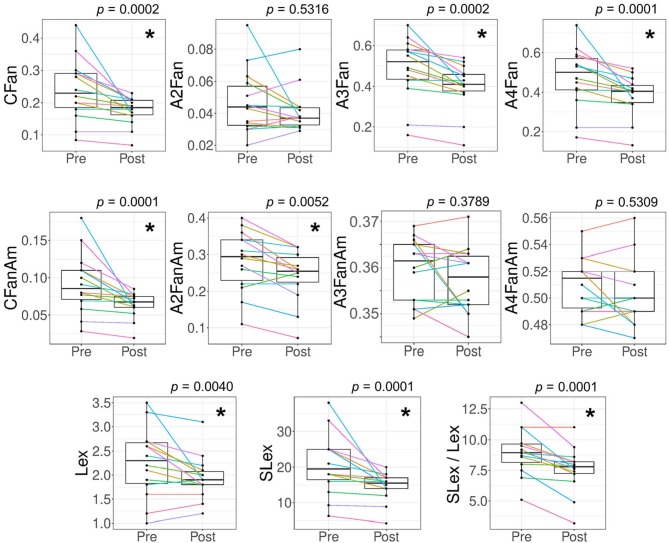
Alteration in features related to antennary fucosylated glycans for colorectal cancer patient samples. Derived traits for glycans were calculated to evaluate antennary fucosylation changes between the 14 pairs of pre-operative (Pre) vs. post-operative (Post) colorectal cancer patient samples. The patient samples were measured with the antennary fucose assay (MALDI-FT-ICR-MS readout). Significant changes were observed for antennary fucosylation in complex *N*-glycans (**CFan**), antennary fucosylation in triantennary glycans (**A3Fan**), antennary fucosylation in tetraantennary glycans (**A4Fan**), α2,3-sialylation per antenna of total antennary fucosylated glycans (**CFanAm**), α2,3-sialylation per antenna of diantennary antennary fucosylated glycans (**A2FanAm**), sialyl-Lewis x abundance (relative area%) in TPNG (**SLex**), Lewis x abundance in TPNG (**Lex**) and the ratio of sialyl-Lewis x to Lewis x abundances in TPNG (**SLex / Lex**). No significant changes were observed for antennary fucosylation in diantennary glycans (**A2Fan**), α2,3-sialylation per antenna of triantennary antennary fucosylated glycans (**A3FanAm**) and α2,3-sialylation per antenna of tetraantennary antennary fucosylated glycans (**A4FanAm**). The *p-*values shown are from a Wilcoxon matched-pairs signed-rank test with confidence level taken as 95%. Multiple-testing correction was performed using a false discovery rate of 1% calculated with the Benjamini and Hochberg method. The *p*-values < 0.0073 are considered significant and are represented with an asterisk (*).

The feasibility to analyze clinically relevant markers of antennary fucosylation with our assay was demonstrated on sample pairs (pre-operative vs. post-operative) of 14 colorectal cancer patients. Glycosylation changes in colorectal cancer patient samples are shown in Figure 7 and Supplementary Figure S31. Details on features and their calculations are shown in Supplementary Table S4. In line with previous findings, we were able to show that post-operative patient samples had a significantly lower total antennary fucosylation on total complex *N*-glycans (CFan) as compared to pre-operative samples (Figure 7). Unlike previously, no significant differences were observed in α2,3-sialylation per antenna of antennary fucosylated triantennary glycans (A3FanAm), but rather a significantly lowered α2,3-sialylation per antenna of antennary fucosylation diantennary glycans (A2FanAm) in post-operative patient samples as compared to pre-operative samples (Figure 7). Since we used only a quarter of the samples, compared to the previous study, missing statistical power provides a simple explanation for missing the sialylation effect on fucosylated triantennary glycans. However, when we focused on the quantitation of sialyl-Lewis x epitopes, it was possible to reproduce the finding. We are able to calculate highly specific derived traits based on antennary fucosylated glycans structures, allowing us to study more specific glycan features. For example, assuming α2,6-sialylated antennae are not fucosylated, there is no preference for fucosylation of α2,3-sialylated or asialylated antennae and multiple fucoses are on different arms, the relative abundances of sialyl-Lewis x can be calculated. This is indeed lowered in the post-operative samples (Figure 7). The discovery of a novel association with A2FanAm is also easily explained by the increased specificity of our assay. The trait is largely composed of compositions representing a mixture of core and antennary fucose isomers before core defucosylation. Hence, in a regular TPNG/TSNG MALDI-MS analysis unrelated variations in core fucosylation would interfere with the detection of changes in antennary fucosylation of diantennary glycans.

The changes in CFan are mainly contributed by the triantennary glycans (A3Fan) and tetraantennary glycans (A4Fan) rather than the diantennary glycans (A2Fan) and bisecting diantennary glycans (A2BFan) (A2BFan shown in Supplementary Figure S31). We did not observe a change in A2Fan and A2BFan. Without core defucosylation, these traits would largely measure core fucosylation (Supplementary Figure S4). Furthermore, we also observed a significantly lowered multiantennary fucosylation (CFm_an) in post-operative patient samples as compared to pre-operative samples (Supplementary Figure S31). Finally, we also approximated the abundance of glycans having Lex or sialyl-Lewis x epitopes in TPNG. sialyl-Lewis x / Lewis x ratio was significantly lowered in post-operative patient samples as compared to pre-operative samples. This change was mainly contributed by a decreased abundance of sialyl-Lewis x in post-operative samples, although the Lewis x abundance was also lowered. This may be associated with a decreased inflammatory state of the recovering patient (Brinkman-van der Linden et al., 1998; Higai et al., 2005).

All studied features, except for Lewis x (*p* = 0.0513), were replicated for the MALDI-TOF-MS measurements of the samples (Supplementary Figure S32). Thus, MALDI-TOF-MS measurements are sufficient to detect many of the clinical changes.

## Conclusion

We developed an assay for the relative quantitation of antennary fucosylation and approximation of Lewis x and sialyl-Lewis x abundances in TPNG, based on high-throughput MALDI-MS analysis. This assay is compatible with high sensitivity and ultrahigh-resolution MALDI-FT-ICR-MS or with MALDI-TOF-MS. In total, 19 antennary fucosylated glycans were relatively quantified with precision and accuracy expectable of a MALDI-MS approach. Furthermore, the assay was applied to measuring biomedically relevant changes in antennary fucosylation in colorectal cancer patients pre vs. post tumor resection. Next to previous findings that could be repeated, despite the reduced sample size, the increased specificity enabled the discovery of novel associations. Additionally, we were able to investigate more specialized features based on antennary fucosylation which would not be possible with regular TPNG analysis. The next steps would include further automatization of the assay and perform a high throughput analysis on a large set of patient samples.

## Data Availability Statement

The datasets generated for this study are available on request to the corresponding author.

## Ethics Statement

The studies involving human participants were reviewed and approved by the pre-operative vs. 45 days post-operative pairs of 14 CRC patient samples were collected as part of a biobank as was previously described (de Vroome et al., 2018). These serum samples were collected between October 2002 and March 2013 by the Leiden University Medical Center (LUMC) Surgical Oncology Biobank. This study was approved by the Medical Ethics Committee of the LUMC and was performed in accordance with the Code of Conduct of the Federation of Medical Scientific Societies in the Netherlands (http://www.federa.org/). The patients/participants provided their written informed consent to participate in this study.

## Author Contributions

OR performed all experiments (except HILIC-FLD-MS measurements) and analyzed all data with the help of DF. OR was supervised by SN for the MALDI-FT-ICR-MS experiments and by JN and GL-K for the CE-ESI-MS/MS experiments, respectively. DF supervised the sample preparation and MALDI-TOF-MS experiments. RG performed the HILIC-FLD-MS experiments supervised by DS. WM and RT managed the colorectal cancer samples. OR and DF designed the experiments, aided by SN, GL-K, and MW. OR, SN, GL-K, and DF drafted the manuscript. All authors contributed to finalizing the manuscript.

### Conflict of Interest

OR, RG, and DS were employed by Ludger Ltd. The remaining authors declare that the research was conducted in the absence of any commercial or financial relationships that could be construed as a potential conflict of interest.
