# Supplementary material for: A Matrix-Assisted Laser Desorption/Ionization—Mass Spectrometry Assay for the Relative Quantitation of Antennary Fucosylated N-Glycans in Human Plasma
Source: Front Chem. 2020 Feb 28;8:138. doi: 10.3389/fchem.2020.00138 (PMC7059190; doi:10.3389/fchem.2020.00138)
Supplement: Supplementary file 1 [file Data_Sheet_1.PDF]

Supplementary Material

1 Assay optimization

All optimization experiments of the assay were measuremented on the MALDI-TOF-MS. Data processing for these experiments was done using the mass list of the negative control (fucosidase untreated TPNG) (Supplementary Table S7). Quantitation of residual core fucosylated glycans (CFGs) allowed the assessment of the completeness of the core defucosylation. Importantly, all identified antennary fucosylated glycans were also included in the mass list of the negative control (fucosidase untreated TPNG).

A minor (3% - 6%) improvement in core defucosylation was achieved by introducing NP-40 in the digestion reaction (see Supplementary Figure S33). NP-40 is a non-ionic detergent, protecting the enzyme from denaturation caused by SDS that is carried over from the glycan release step. The digestion efficiency was improved to the same extent for all the concentrations of NP-40 tested. Hence, 1% (v/v%) of NP-40 was chosen for further experiments as it was mid-range of the values tested. Using lower amounts of released TPNG, further reduced core fucosidase consumption. A 10 times dilution of the released TPNG still allowed for a quantitation on MALDI-TOF-MS while allowing for a 2 fold lower consumption of fucosidase (see Supplementary Figure S34). However, for these conditions, matrix clusters interfered with the quantification of lower mass glycans (m/z 1000 to 2000) in the MALDI-FT-ICR-MS spectra. By lowering the Na+ ion concentration in the MALDI matrix of superDHB to 0.1 M sodium hydroxide, the abundance of these matrix clusters was reduced by about 4 folds (see Supplementary Figures S35 and S36). The observation of matrix clusters in the MALDI-FT-ICR-MS spectra and not in the MALDI-TOF-MS spectra, can be explained by the higher pressure in the source of the MALDI-FT-ICR-MS. This can allow for a quicker dissipation of energy from the ionized clusters in the gas phase and thus counteracting their de-clustering (Karas and Kruger, 2003). Importantly, no proton or potassium adducts of the glycans were observed after reduction from 1 M to 0.1 M sodium hydroxide.

Karas, M., and Kruger, R. (2003). Ion formation in MALDI: the cluster ionization mechanism. *Chem Rev* 103(2), 427-440. doi: 10.1021/cr010376a.

2 Supplementary Figures

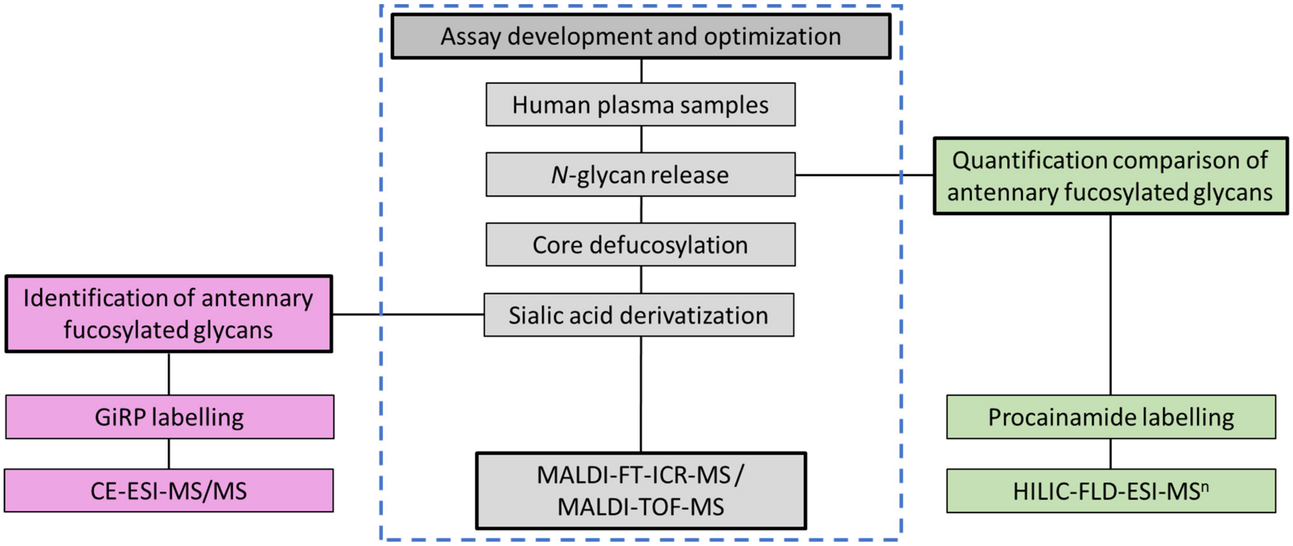

Supplementary Figure S1. Flowchart of analytical methods used for assay development. MALDI-FT-ICR-MS and MALDI-TOF-MS were used in the assay for sample measurements. The quantitation of antennary fucosylation in TPNG by the assay was compared with TPNG analysis performed on a HILIC-FLD-MS^n system. CE-ESI-MS/MS was used for identification of antennary fucosylated glycans by CID fragmentation.

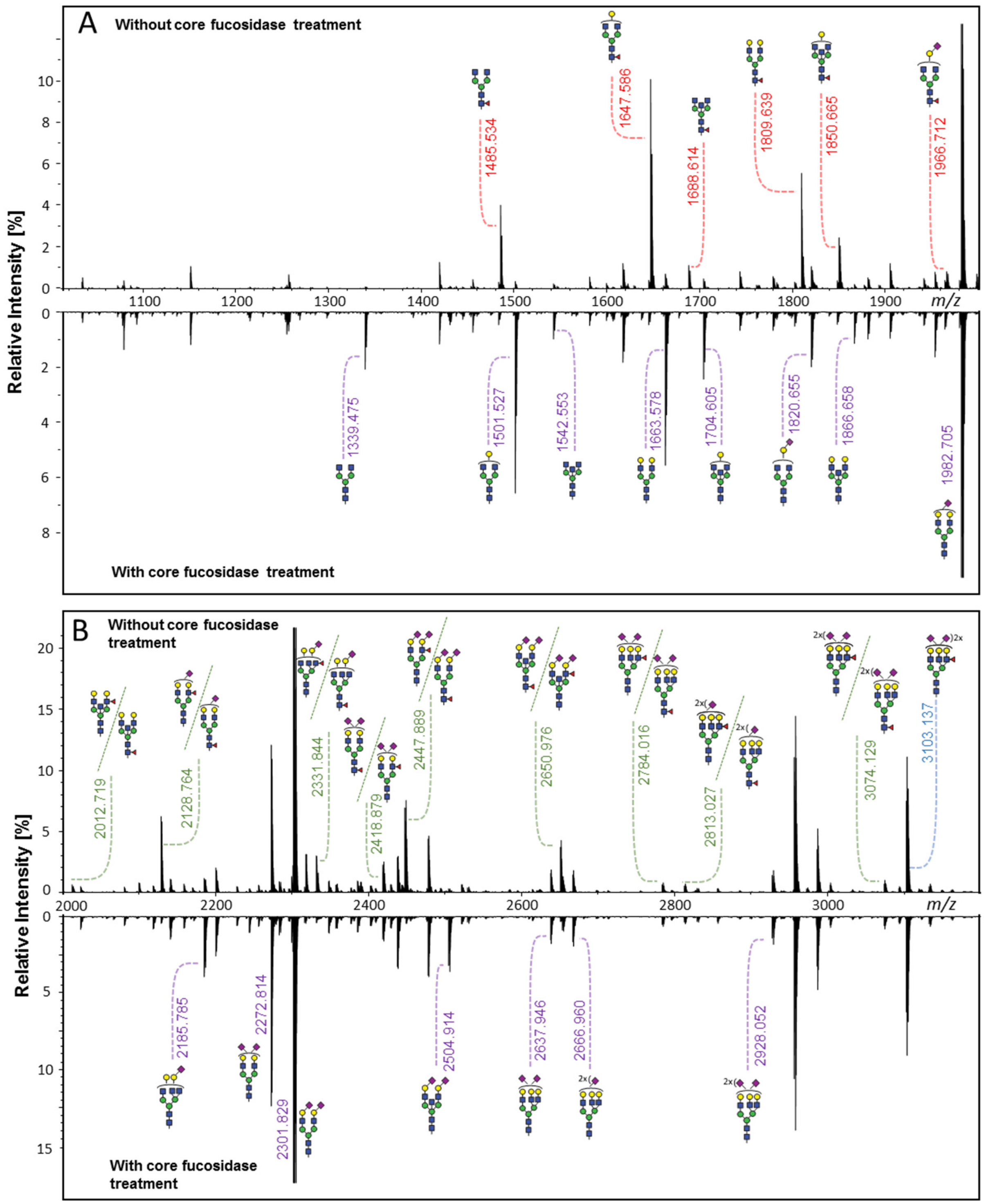
A
Without core fucosidase treatment
1485.534
1647.586
1688.614
1809.639
1850.665
1966.712
1339.475
1501.527
1542.553
1663.578
1704.605
1820.655
1866.658
1982.705
With core fucosidase treatment
Relative Intensity [%]
m/z
B
Without core fucosidase treatment
2012.719
2128.764
2331.844
2418.879
2447.889
2650.976
2784.016
2813.027
3074.129
3103.137
2185.785
2272.814
2301.829
2504.914
2637.946
2666.960
2928.052
With core fucosidase treatment
Relative Intensity [%]
m/z

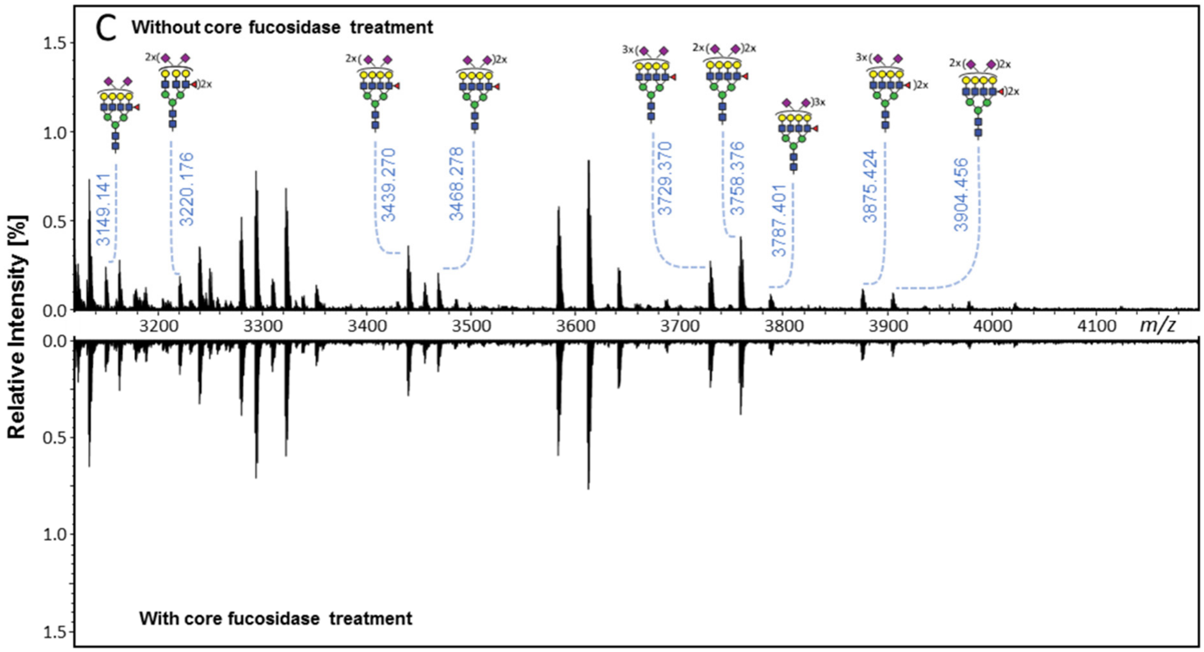

Supplementary Figure S2. Identification of core fucosylated glycans and antennary fucosylated glycans by exoglycosidase and MALDI-FT-ICR-MS. The TPNG profile without core fucosidase treatment was compared to the obtained profile after treatment, within ranges of **(A)** *m/z* 1010 to 2000, **(B)** *m/z* 2000 to 3200 and **(C)** *m/z* 3120 to 4200. Core fucosylated glycans [red *m/z* values] are converted to their corresponding afucosylated glycans [purple *m/z* values], upon core fucosidase treatment. Only the antennary fucosylated glycans [blue *m/z* values] and the antennary fucose isomers of the mixed fucose isomeric glycans (core or antennary fucosylation) [green *m/z* values] remain after core fucosidase treatment. All *m/z* values of annotated glycans belong to $[M + Na]^+$ ions. The descriptions of the glycan cartoons are shown in Figure 1.

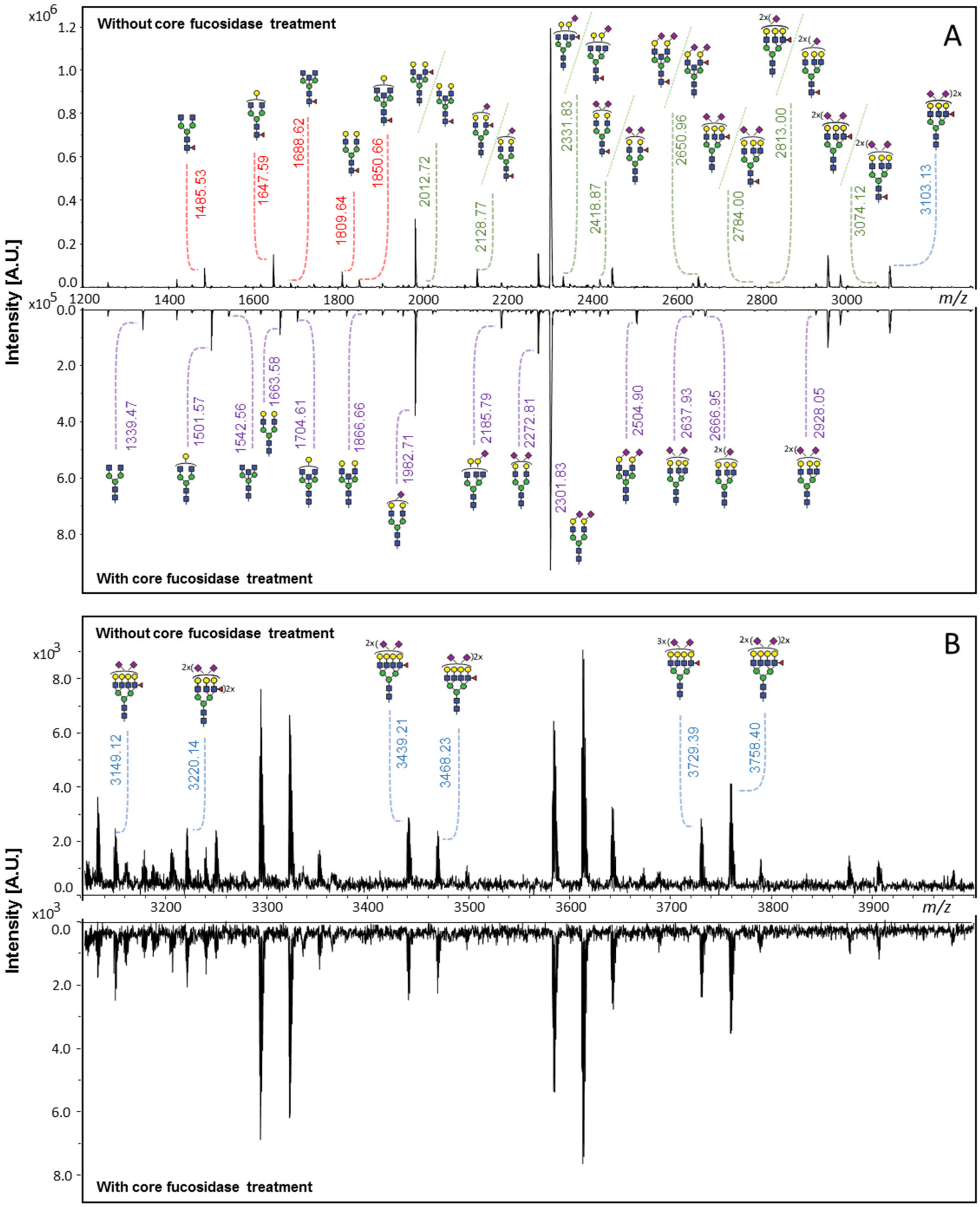

Supplementary Figure S3. Identification of core fucosylated glycans and antennary fucosylated glycans by exoglycosidase and MALDI-TOF-MS. The TPNG profile without core fucosidase treatment was compared to the obtained profile after treatment, within ranges of **(A)** *m/z* 1200 to 3200 and **(B)** *m/z* 3120 to 4000. Core fucosylated glycans [red *m/z* values] are converted to their corresponding afucosylated glycans [purple *m/z* values], upon core fucosidase treatment. Only the antennary fucosylated glycans [blue *m/z* values] and the antennary fucose isomers of the mixed fucose isomeric glycans (core or antennary fucosylation) [green *m/z* values] remain after core fucosidase treatment. All *m/z* values of annotated glycans belong to $[M + Na]^+$ ions. The descriptions of the glycan cartoons are shown in Figure 1.

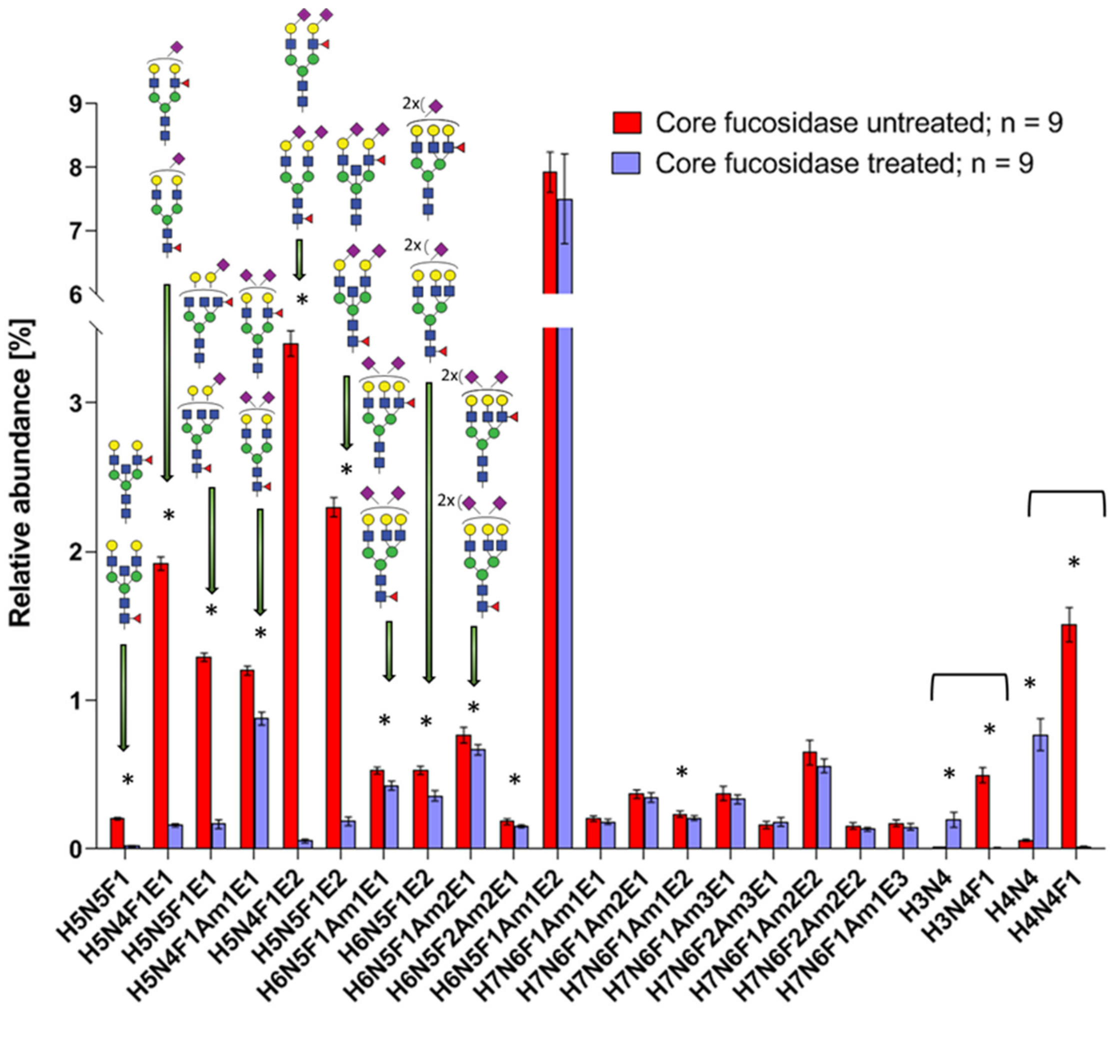

Supplementary Figure S4. Identification of antennary fucosylated or core fucosylated isomeric glycans in TPNG. The relative abundance of the remaining antennary fucosylated glycans after core fucosidase treatment [blue] are compared to TPNG analysis without fucosidase treatment [red]. Glycans that show a significant difference in abundance, due to the depletion of core fucosylated isomers, are marked with an asterix (*). The cartoons represent monofucosylated isomeric glycans in TPNG (fucosidase untreated) that can be either core fucosylated or antennary fucosylated. The glycans H3N4F1 and H4N4F1, and their corresponding afucosylated forms, serve as quality check for depletion of core fucosylation. The error bars show standard deviation of the mean (n = 9) that were measured on the MALDI-FT-ICR-MS. Statistical analysis was performed by multiple t test (α <= 5%) and false discovery rate (FDR = 1%) test of Benjamini and Hochberg. The *p* values < 0.007 are considered significant (*). The descriptions of the glycan cartoons are as shown in Figure 1. The glycan compositions are described in Figure 6.

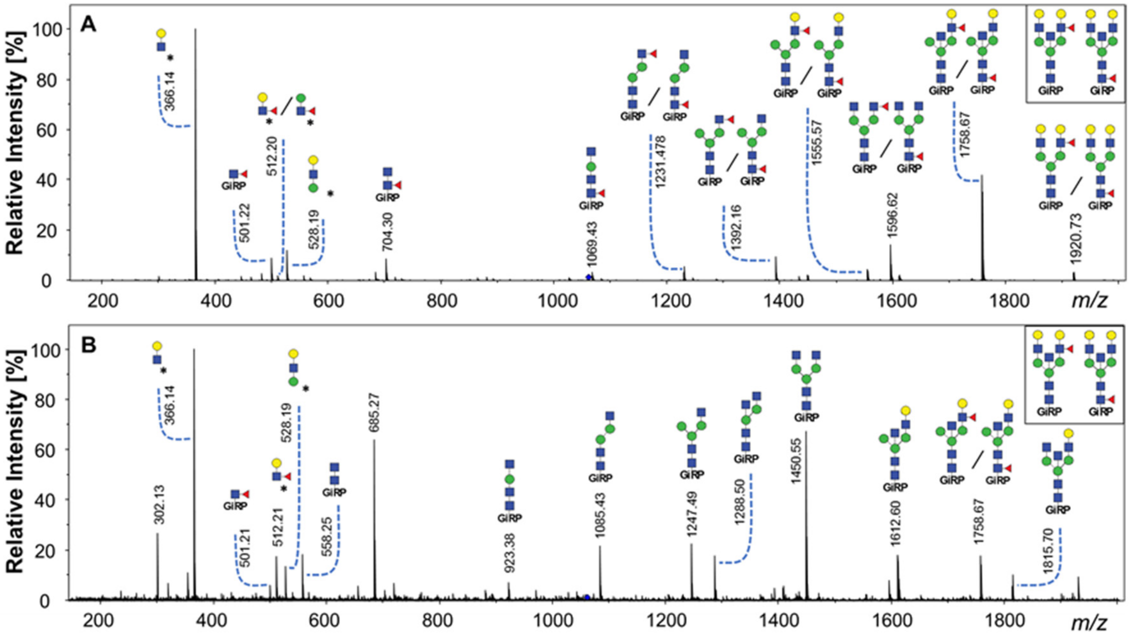

Supplementary Figure S5. CE-ESI-MS/MS spectra of H5N5F1 from sialic acid derivatized and GiRP labelled TPNG. The $[M+H]^{2+}$ ion of H5N5F1 (*m/z* 1062.405) was fragmented from TPNG **(A)** without core fucosidase treatment and **(B)** with core fucosidase treatment. The description of the glycan cartoons are as described in Figure 1. GiRP represents the Girard's reagent P label; * represents an oxonium ion.

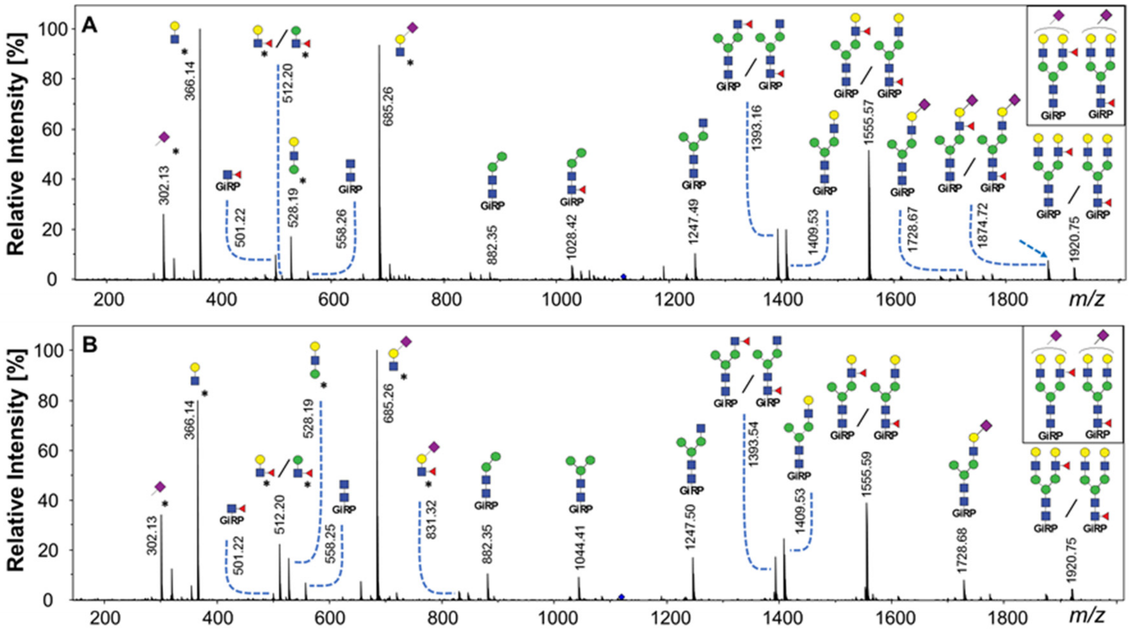

Supplementary Figure S6. CE-ESI-MS/MS spectra of H5N4F1E1 from sialic acid derivatized and GiRP labelled TPNG. The $[M+H]^{2+}$ ion of H5N4F1E1 (*m/z* 1120.429) was fragmented from TPNG **(A)** without core fucosidase treatment and **(B)** with core fucosidase treatment. The description of the glycan cartoons are as described in Figure 1. GiRP represents the Girard's reagent P label; * represents an oxonium ion.

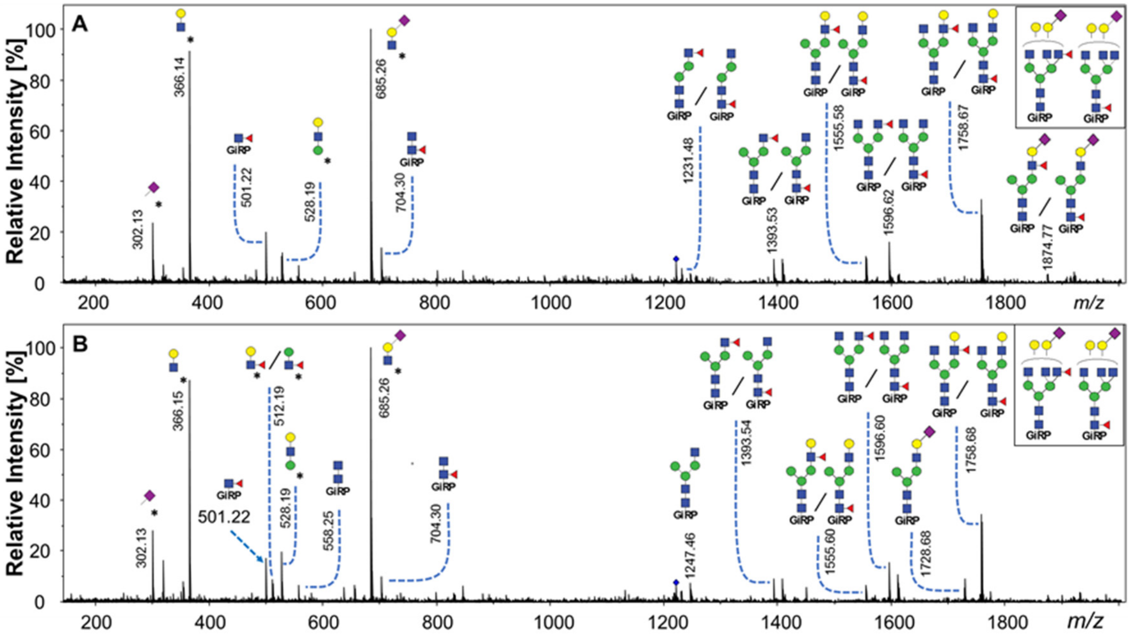

Supplementary Figure S7. CE-ESI-MS/MS spectra of H5N5F1E1 from sialic acid derivatized and GiRP labelled TPNG. The $[M+H]^{2+}$ ion of H5N5F1E1 (*m/z* 1221.971) was fragmented from TPNG **(A)** without core fucosidase treatment and **(B)** with core fucosidase treatment. The description of the glycan cartoons are as described in Figure 1. GiRP represents the Girard's reagent P label; * represents an oxonium ion.

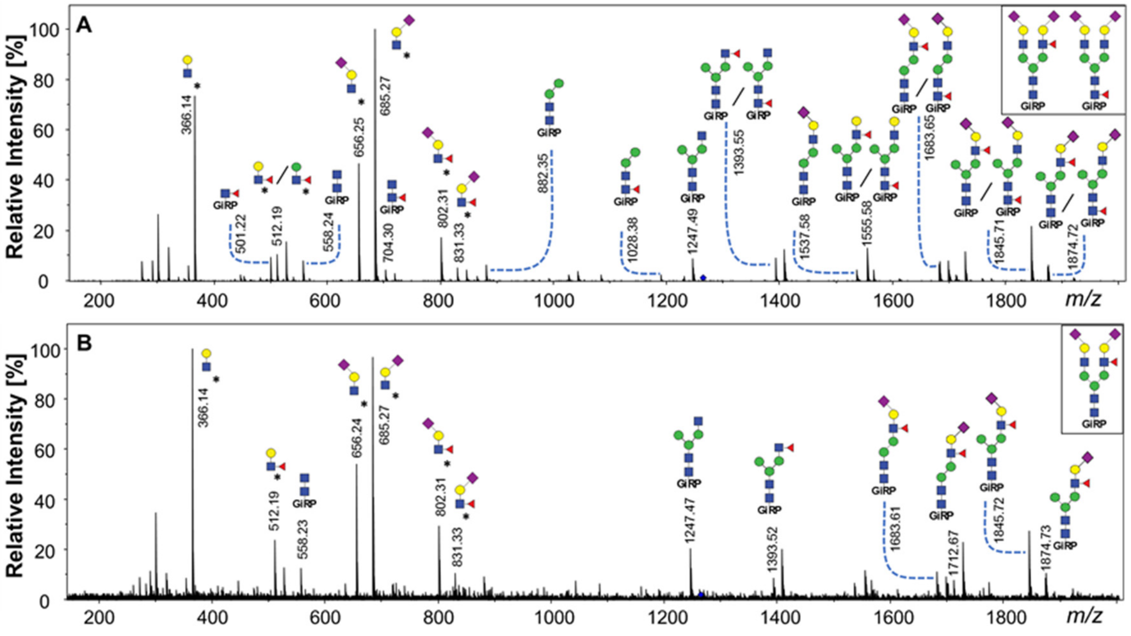

Supplementary Figure S8. CE-ESI-MS/MS spectra of H5N4F1Am1E1 from sialic acid derivatized and GiRP labelled TPNG. The $[M+H]^{2+}$ ion of H5N4F1Am1E1 (*m/z* 1265.485) was fragmented from TPNG **(A)** without core fucosidase treatment and **(B)** with core fucosidase treatment. The description of the glycan cartoons are as described in Figure 1. GiRP represents the Girard's reagent P label; * represents an oxonium ion.

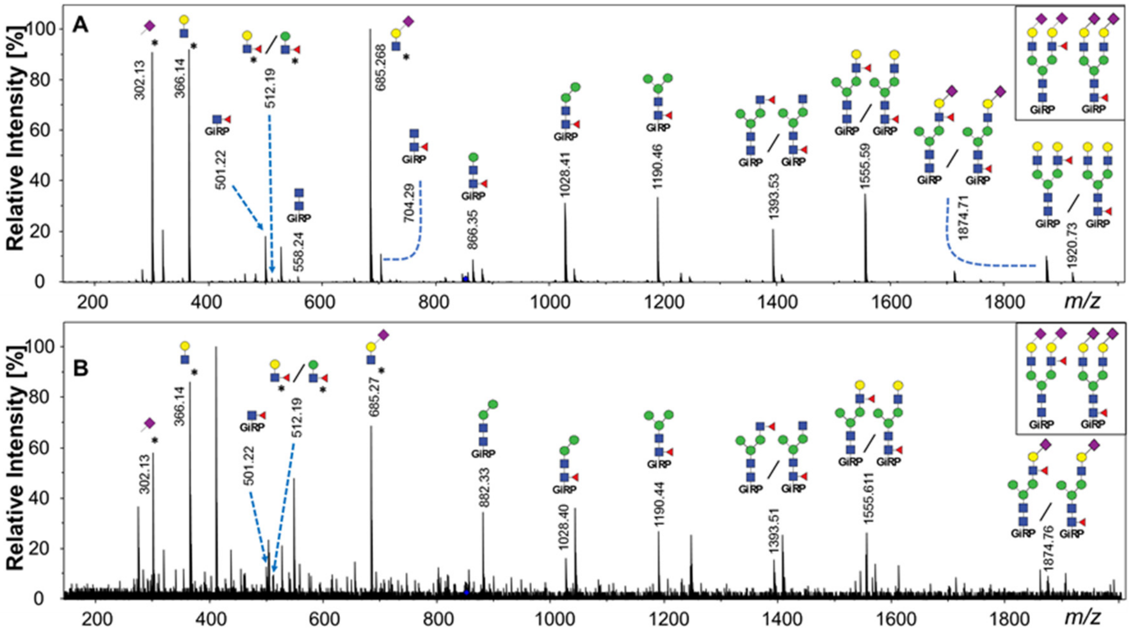

Supplementary Figure S9. CE-ESI-MS/MS spectra of H5N4F1E2 from sialic acid derivatized and GiRP labelled TPNG. The $[M+2H]^{3+}$ ion of H5N4F1E2 (*m/z* 853.660) was fragmented from TPNG **(A)** without core fucosidase treatment and **(B)** with core fucosidase treatment. The description of the glycan cartoons are as described in Figure 1. GiRP represents the Girard's reagent P label; * represents an oxonium ion.

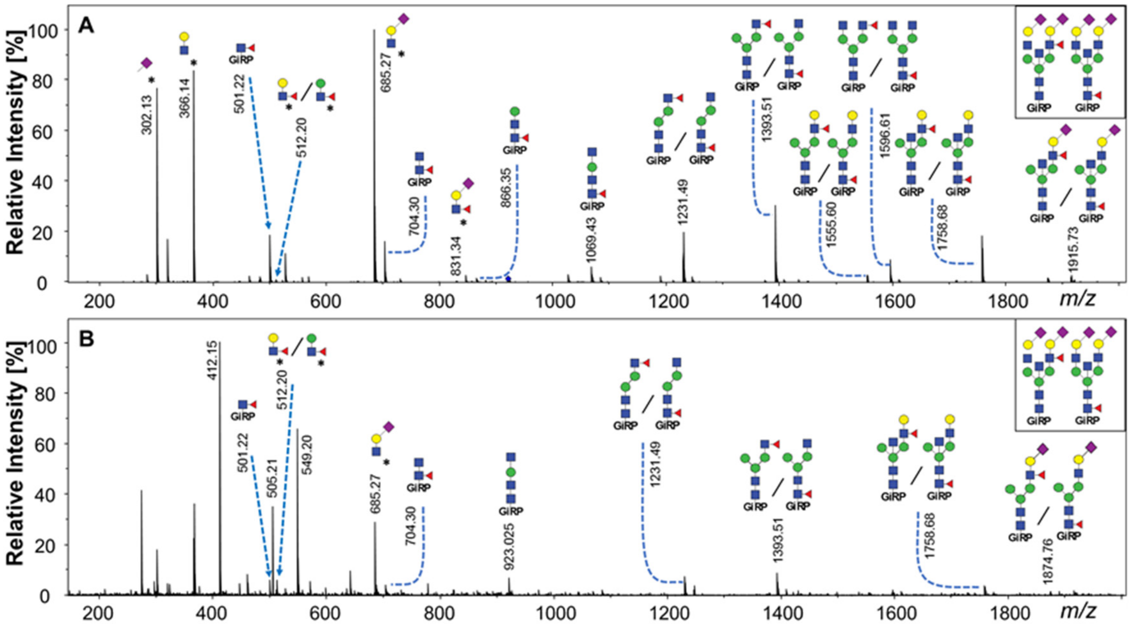

Supplementary Figure S10. CE-ESI-MS/MS spectra of H5N5F1E2 from sialic acid derivatized and GiRP labelled TPNG. The $[M+2H]^{3+}$ ion of H5N5F1E2 (*m/z* 921.653) [second isotope] was fragmented from TPNG **(A)** without core fucosidase treatment and **(B)** with core fucosidase treatment. The description of the glycan cartoons are as described in Figure 1. GiRP represents the Girard's reagent P label; * represents an oxonium ion.

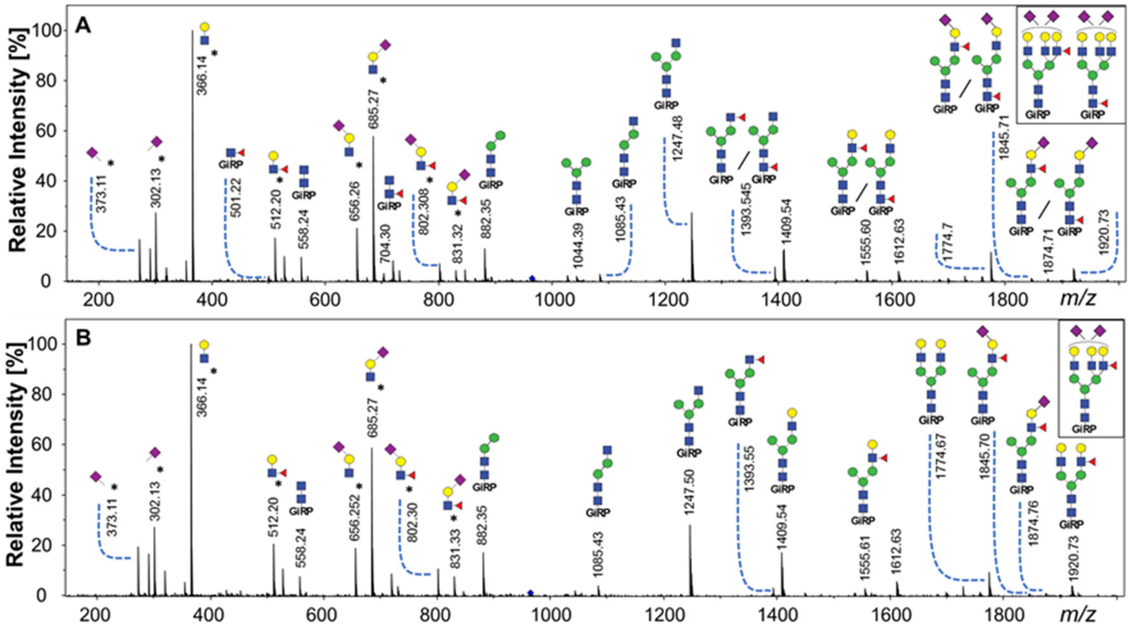

Supplementary Figure S11. CE-ESI-MS/MS spectra of H6N5F1Am1E1 from sialic acid derivatized and GiRP labelled TPNG. The $[M+2H]^{3+}$ ion of H6N5F1Am1E1 (*m/z* 966.037) [second isotope] was fragmented from TPNG **(A)** without core fucosidase treatment and **(B)** with core fucosidase treatment. The description of the glycan cartoons are as described in Figure 1. GiRP represents the Girard's reagent P label; * represents an oxonium ion.

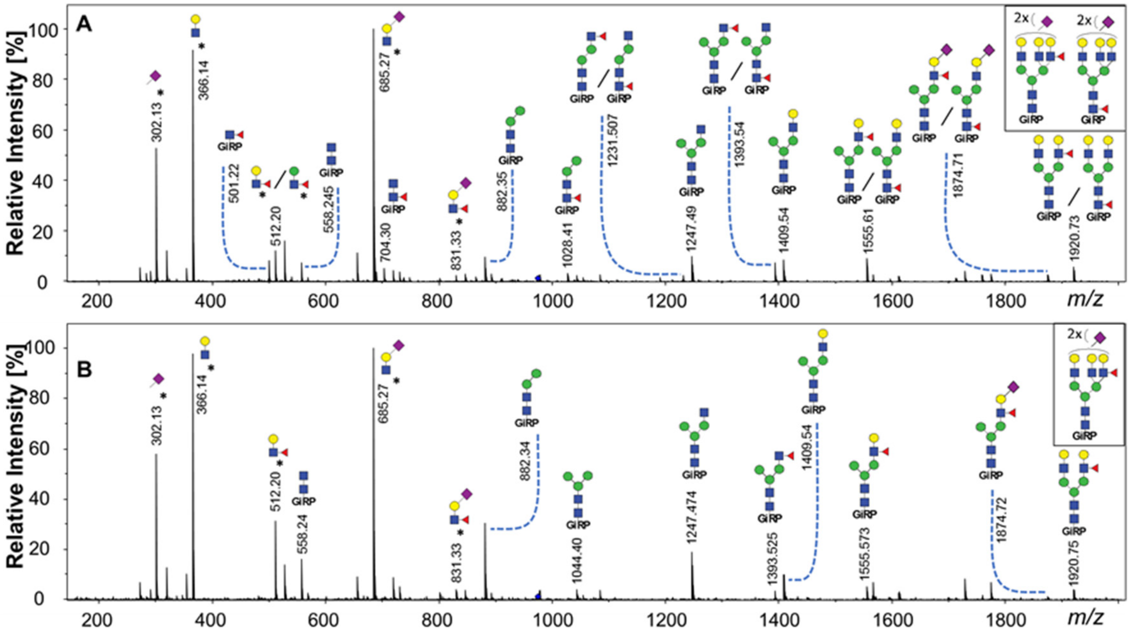

Supplementary Figure S12. CE-ESI-MS/MS spectra of H6N5F1E2 from sialic acid derivatized and GiRP labelled TPNG. The $[M+2H]^{3+}$ ion of H6N5F1E2 (*m/z* 975.707) [second isotope] was fragmented from TPNG **(A)** without core fucosidase treatment and **(B)** with core fucosidase treatment. The description of the glycan cartoons are as described in Figure 1. GiRP represents the Girard's reagent P label; * represents an oxonium ion.

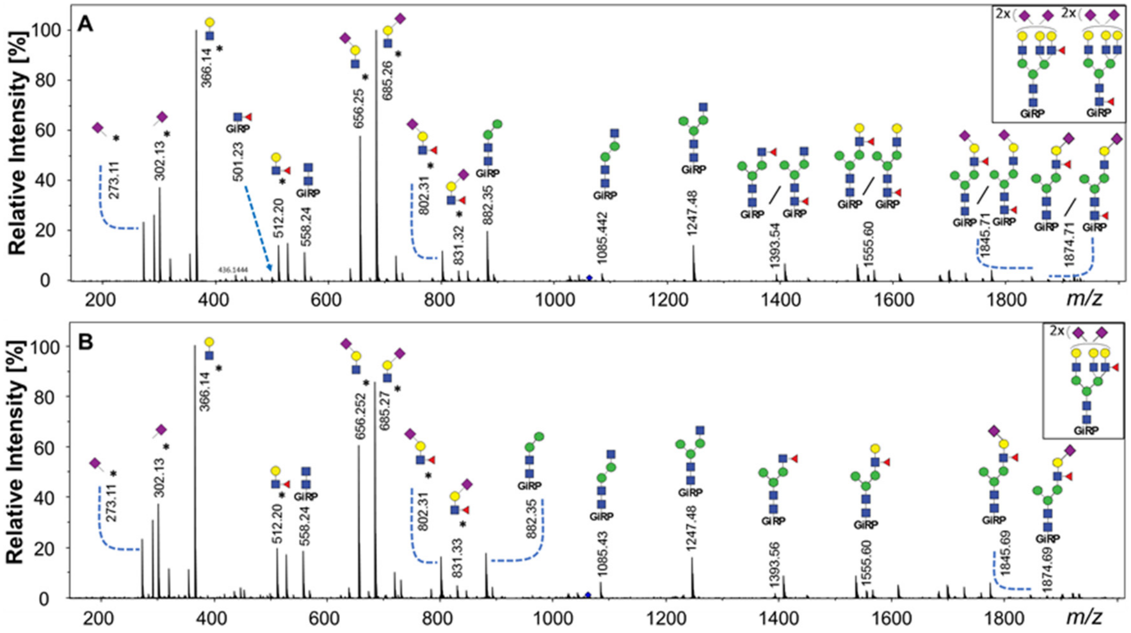

Supplementary Figure S13. CE-ESI-MS/MS spectra of H6N5F1Am2E1 from sialic acid derivatized and GiRP labelled TPNG. The $[M+2H]^{3+}$ ion of H6N5F1Am2E1 (*m/z* 1062.741) [second isotope] was fragmented from TPNG **(A)** without core fucosidase treatment and **(B)** with core fucosidase treatment. The description of the glycan cartoons are as described in Figure 1. GiRP represents the Girard's reagent P label; * represents an oxonium ion.

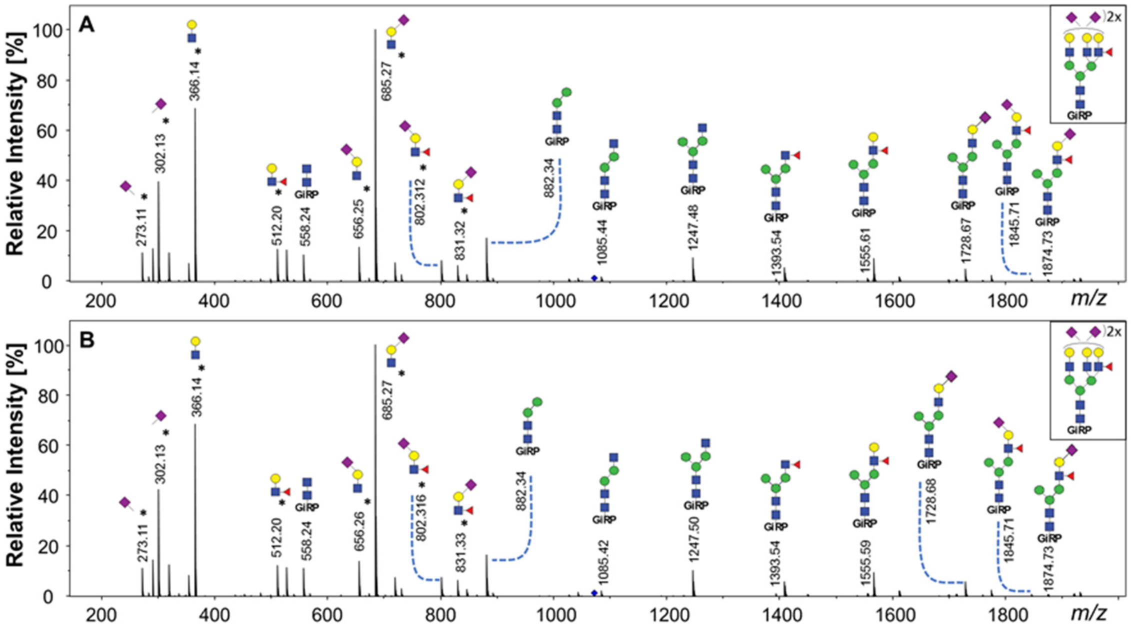

Supplementary Figure S14. CE-ESI-MS/MS spectra of H6N5F1Am1E2 from sialic acid derivatized and GiRP labelled TPNG. The $[M+2H]^{3+}$ ion of H6N5F1Am1E2 (*m/z* 1072.418) [second isotope] was fragmented from TPNG **(A)** without core fucosidase treatment and **(B)** with core fucosidase treatment. The description of the glycan cartoons are as described in Figure 1. GiRP represents the Girard's reagent P label; * represents an oxonium ion.

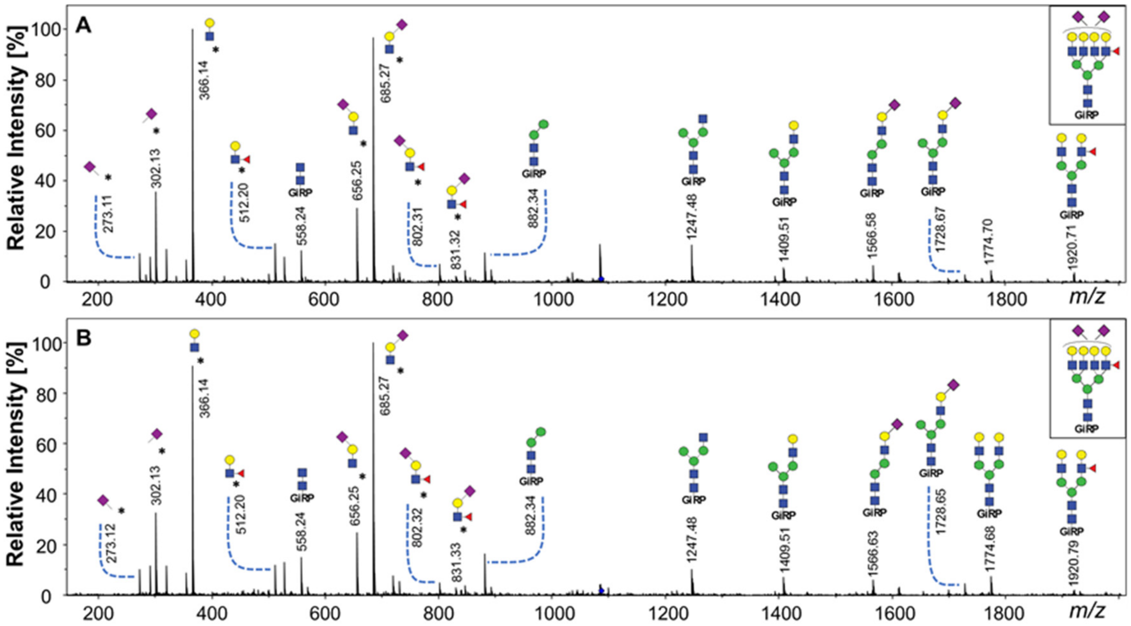

Supplementary Figure S15. CE-ESI-MS/MS spectra of H7N6F1Am1E1 from sialic acid derivatized and GiRP labelled TPNG. The $[M+2H]^{3+}$ ion of H7N6F1Am1E1 (*m/z* 1087.416) [second isotope] was fragmented from TPNG **(A)** without core fucosidase treatment and **(B)** with core fucosidase treatment. The description of the glycan cartoons are as described in Figure 1. GiRP represents the Girard's reagent P label; * represents an oxonium ion.

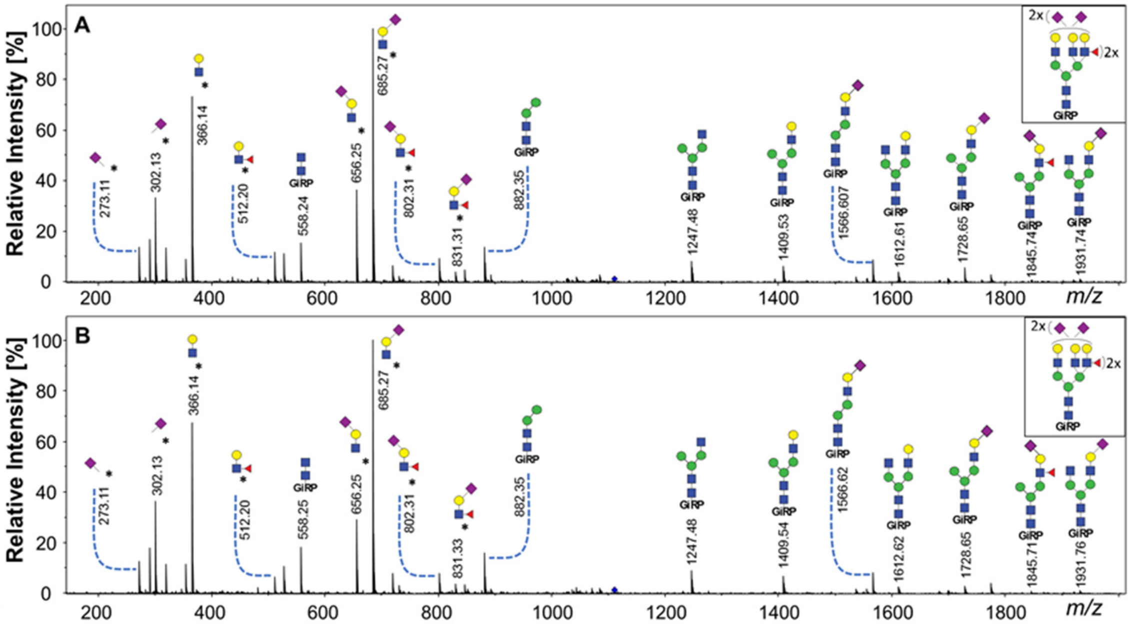

Supplementary Figure S16. CE-ESI-MS/MS spectra of H6N5F2Am2E1 from sialic acid derivatized and GiRP labelled TPNG. The $[M+2H]^{3+}$ ion of H6N5F2Am2E1 (*m/z* 1111.411) [second isotope] was fragmented from TPNG **(A)** without core fucosidase treatment and **(B)** with core fucosidase treatment. The description of the glycan cartoons are as described in Figure 1. GiRP represents the Girard's reagent P label; * represents an oxonium ion.

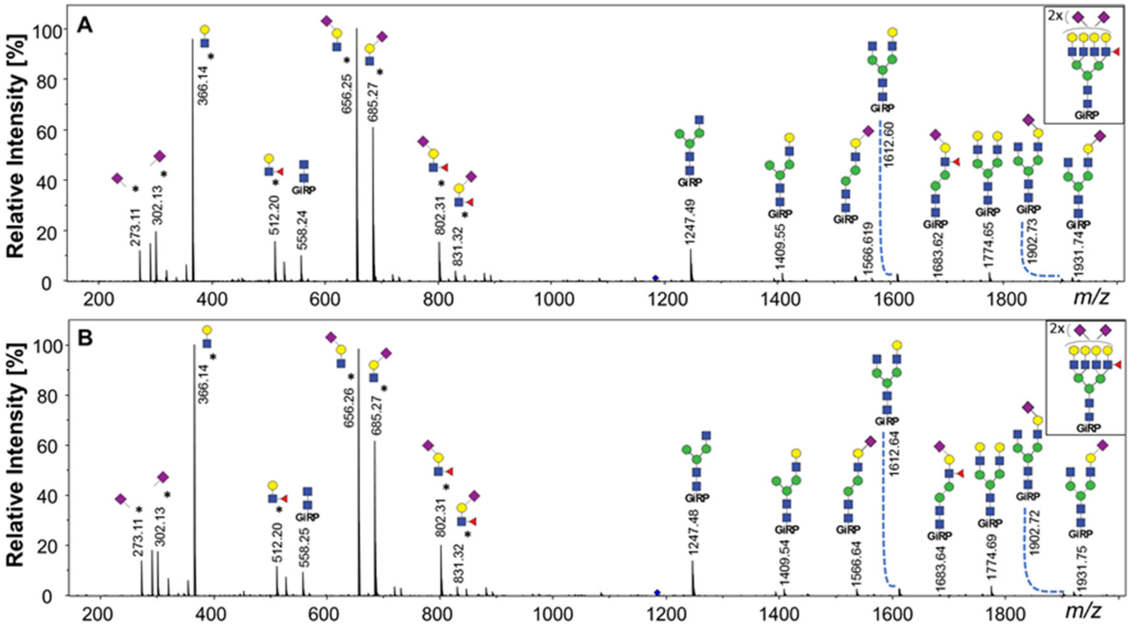

Supplementary Figure S17. CE-ESI-MS/MS spectra of H7N6F1Am2E1 from sialic acid derivatized and GiRP labelled TPNG. The $[M+2H]^{3+}$ ion of H7N6F1Am2E1 (*m/z* 1184.456) [second isotope] was fragmented from TPNG **(A)** without core fucosidase treatment and **(B)** with core fucosidase treatment. The description of the glycan cartoons are as described in Figure 1. GiRP represents the Girard's reagent P label; * represents an oxonium ion.

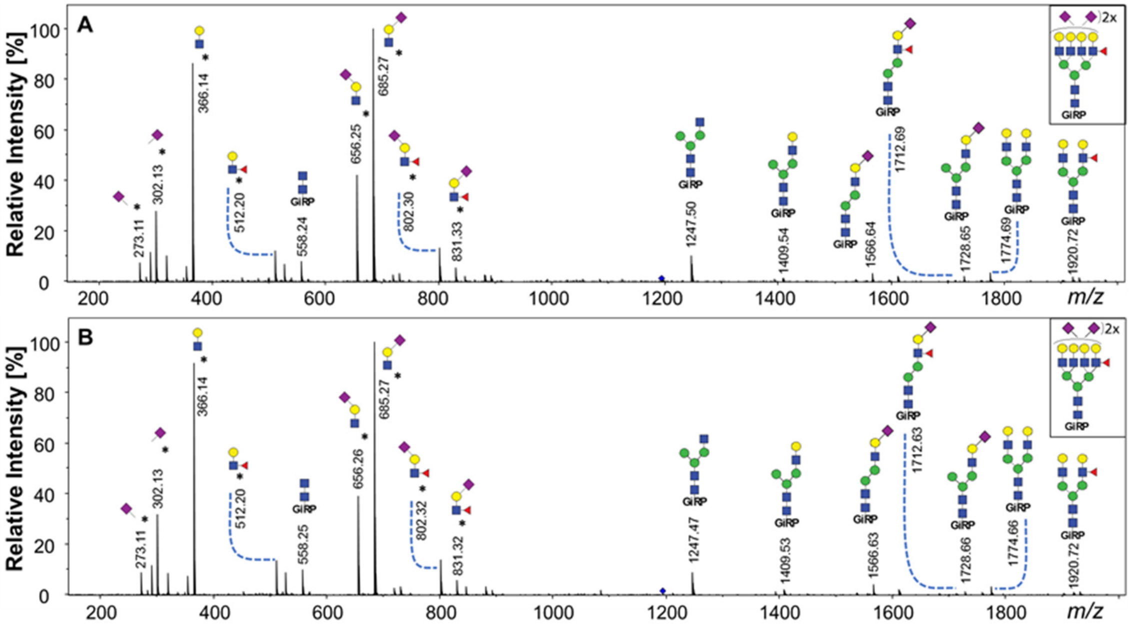

Supplementary Figure S18. CE-ESI-MS/MS spectra of H7N6F1Am1E2 from sialic acid derivatized and GiRP labelled TPNG. The $[M+2H]^{3+}$ ion of H7N6F1Am1E2 (*m/z* 1194.128) [second isotope] was fragmented from TPNG **(A)** without core fucosidase treatment and **(B)** with core fucosidase treatment. The description of the glycan cartoons are as described in Figure 1. GiRP represents the Girard's reagent P label; * represents an oxonium ion.

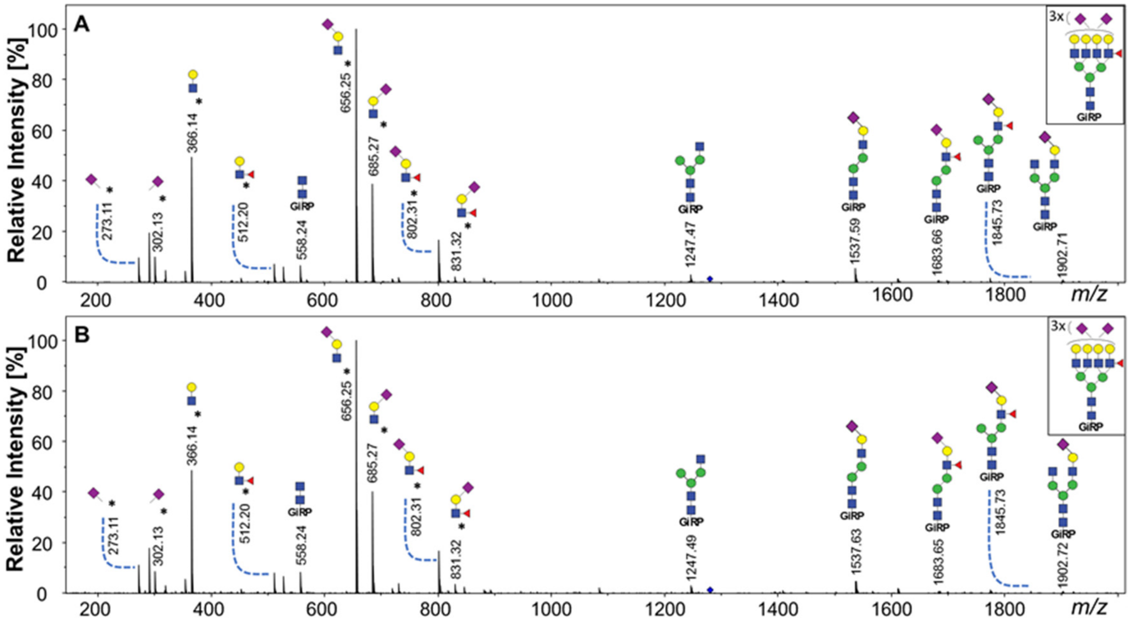

Supplementary Figure S19. CE-ESI-MS/MS spectra of H7N6F1Am3E1 from sialic acid derivatized and GiRP labelled TPNG. The $[M+2H]^{3+}$ ion of H7N6F1Am3E1 (*m/z* 1281.159) [second isotope] was fragmented from TPNG **(A)** without core fucosidase treatment and **(B)** with core fucosidase treatment. The description of the glycan cartoons are as described in Figure 1. GiRP represents the Girard's reagent P label; * represents an oxonium ion.

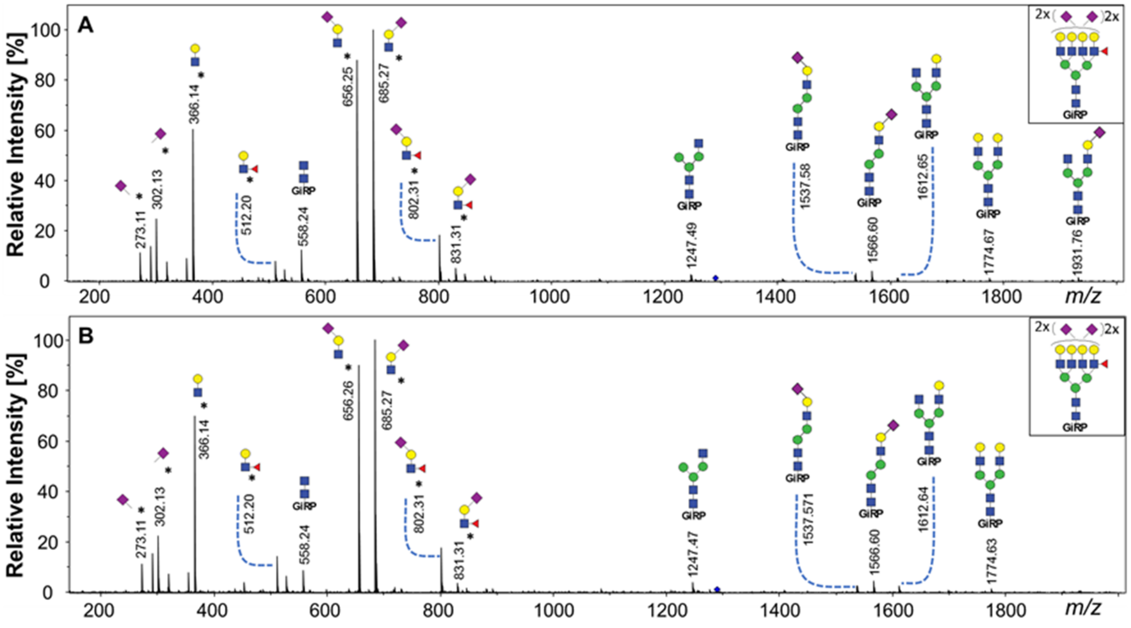

Supplementary Figure S20. CE-ESI-MS/MS spectra of H7N6F1Am2E2 from sialic acid derivatized and GiRP labelled TPNG. The $[M+2H]^{3+}$ ion of H7N6F1Am2E2 (*m/z* 1290.834) [second isotope] was fragmented from TPNG **(A)** without core fucosidase treatment and **(B)** with core fucosidase treatment. The description of the glycan cartoons are as described in Figure 1. GiRP represents the Girard's reagent P label; * represents an oxonium ion.

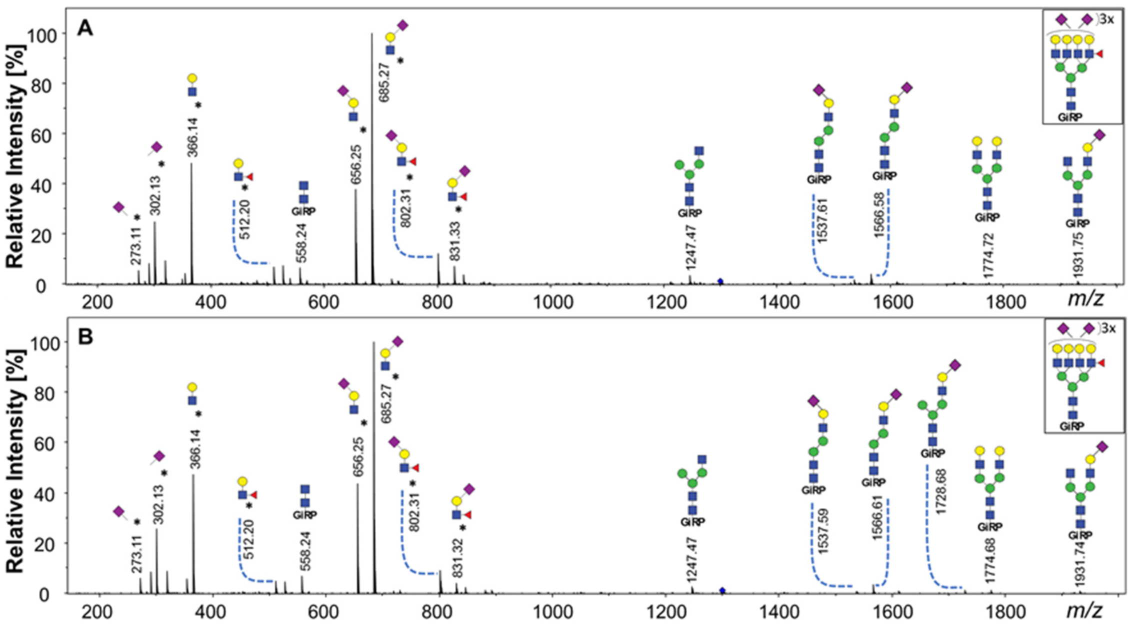

Supplementary Figure S21. CE-ESI-MS/MS spectra of H7N6F1Am1E3 from sialic acid derivatized and GiRP labelled TPNG. The $[M+2H]^{3+}$ ion of H7N6F1Am1E3 (*m/z* 1300.505) [second isotope] was fragmented from TPNG **(A)** without core fucosidase treatment and **(B)** with core fucosidase treatment. The description of the glycan cartoons are as described in Figure 1. GiRP represents the Girard's reagent P label; * represents an oxonium ion.

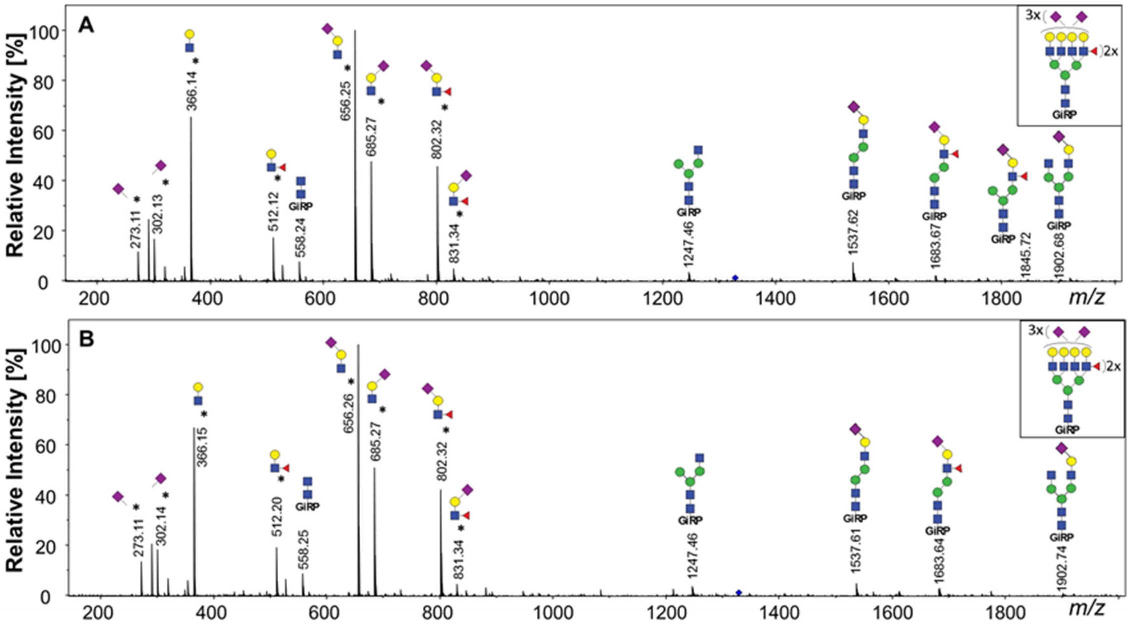

Supplementary Figure S22. CE-ESI-MS/MS spectra of H7N6F2Am3E1 from sialic acid derivatized and GiRP labelled TPNG. The $[M+2H]^{3+}$ ion of H7N6F2Am3E1 (*m/z* 1329.846) [second isotope] was fragmented from TPNG **(A)** without core fucosidase treatment and **(B)** with core fucosidase treatment. The description of the glycan cartoons are as described in Figure 1. GiRP represents the Girard's reagent P label; * represents an oxonium ion.

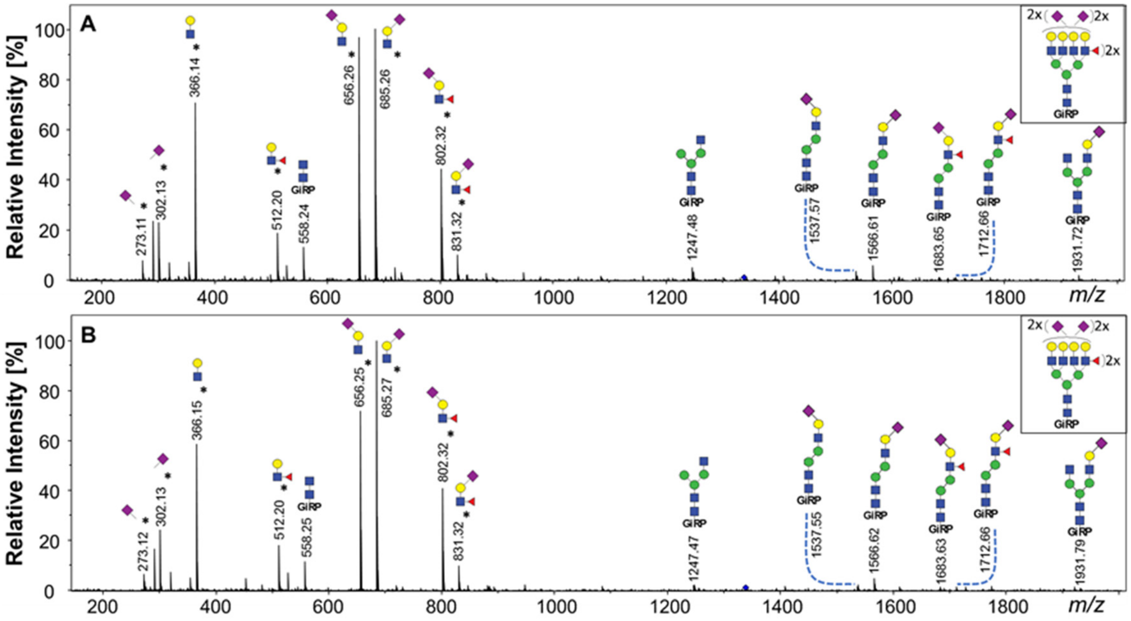

Supplementary Figure S23. CE-ESI-MS/MS spectra of H7N6F2Am2E2 from sialic acid derivatized and GiRP labelled TPNG. The $[M+2H]^{3+}$ ion of H7N6F2Am2E2 (*m/z* 1339.520) [second isotope] was fragmented from TPNG **(A)** without core fucosidase treatment and **(B)** with core fucosidase treatment. The description of the glycan cartoons are as described in Figure 1. Gi GiRP represents the Girard's reagent P label and ; * represents an oxonium ion.

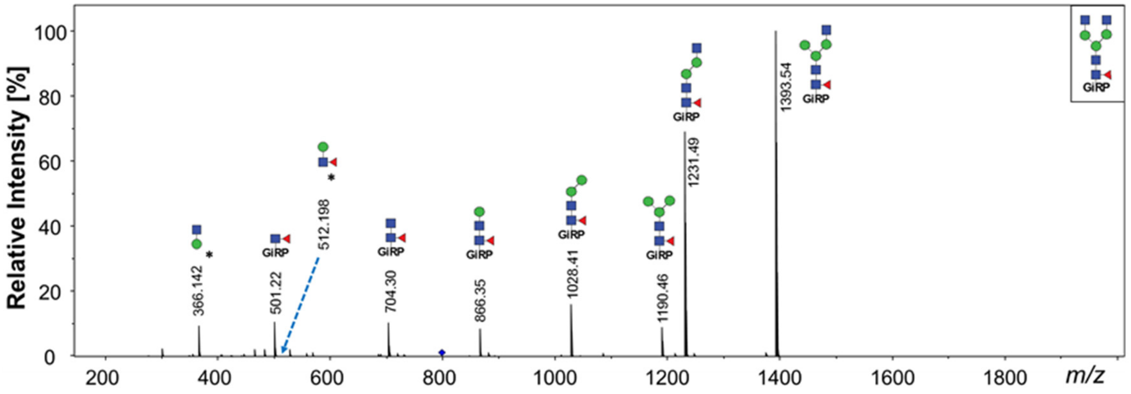

Supplementary Figure S24. CE-ESI-MS/MS spectra of H3N4F1 from sialic acid derivatized and GiRP labelled TPNG. The $[M+H]^{2+}$ ion of H3N4F1 (*m/z* 798.810) was fragmented from TPNG **(A)** without core fucosidase treatment and **(B)** with core fucosidase treatment. The description of the glycan cartoons are as described in Figure 1. GiRP represents the Girard's reagent P label; * represents an oxonium ion.

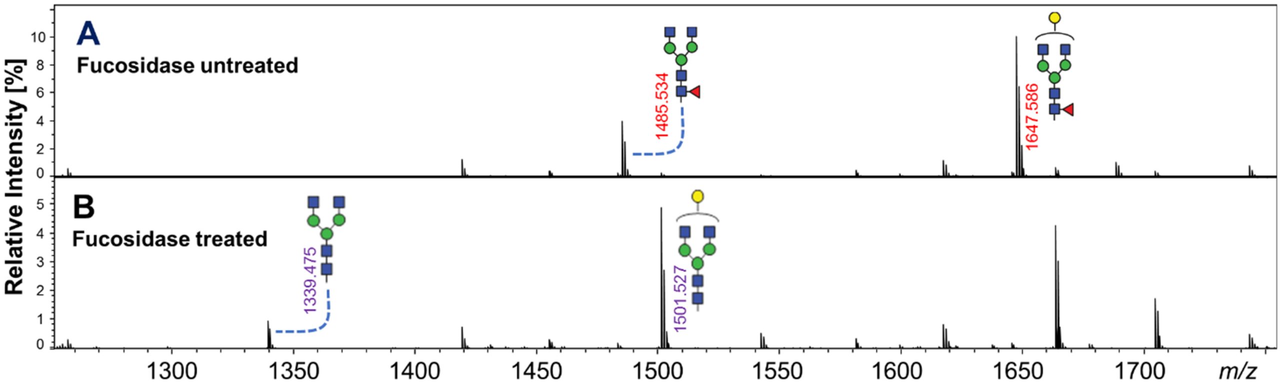

Supplementary Figure S25. Quality check for depletion of core fucosylation in the antennary fucose assay. The assay was measured with MALDI-FT-ICR-MS. The core fucosylated glycans H3N4F1 and H4N4F1 that are observed in **(A)** fucosidase untreated TPNG, are not observed after treatment with **(B)** core fucosidase. Core fucosylated glycans [red *m/z* values] are converted to their corresponding afucosylated glycans [purple *m/z* values], on core fucosidase treatment. The descriptions of the glycan cartoons can be found in Figure 1.

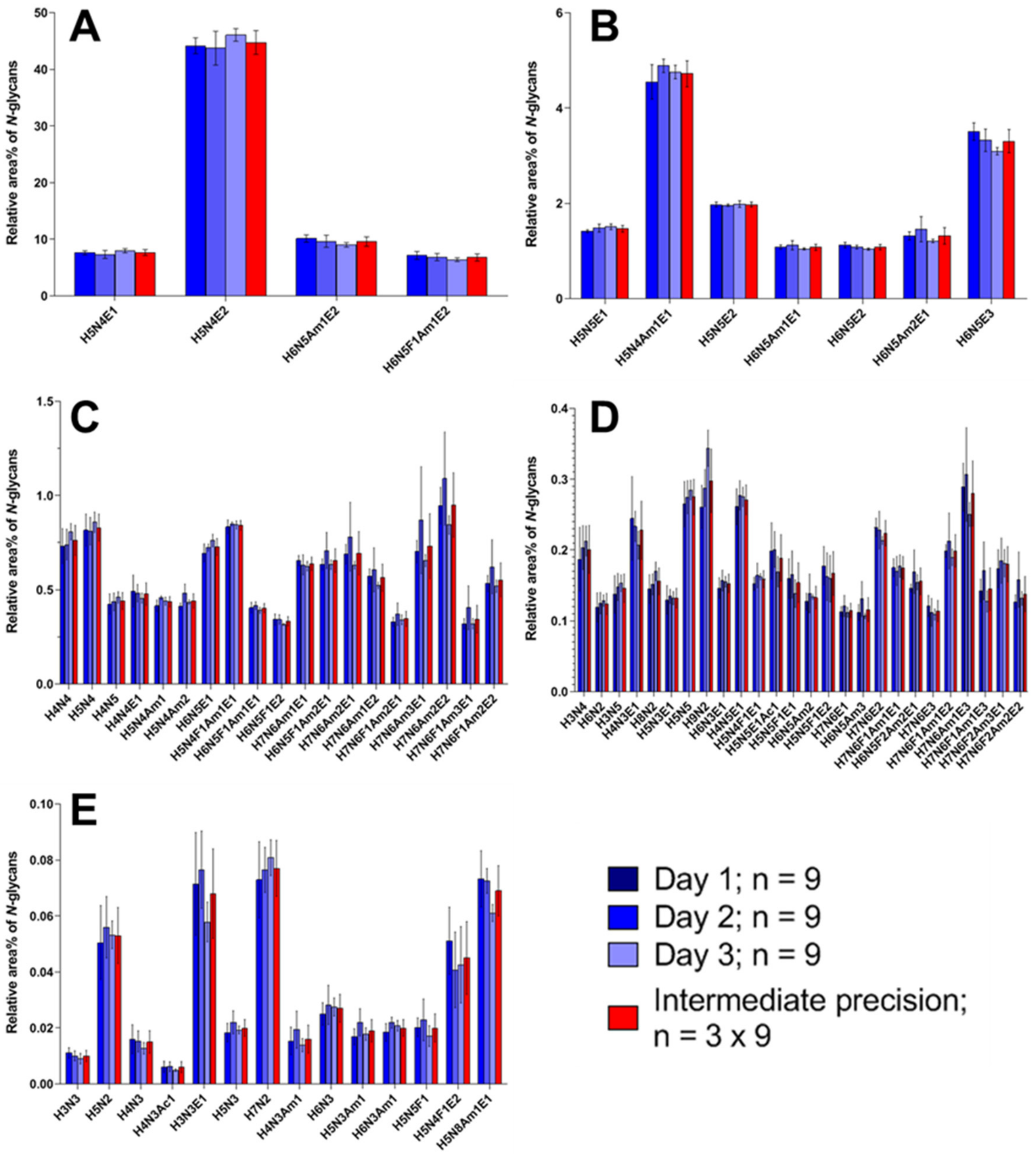

Supplementary Figure S26. Intermediate precision of sample preparation and MADLI-FTICR-MS measurements for human TPNG using the antennary fucose assay. The 70 quantified glycans are sorted according to their relative area (%) **(A)** > 5%, **(B)** 5% to 1%, **(C)** 1% to 0.3%, **(D)** 0.3% to 0.1%, and **(E)** < 0.1%. The error bars show standard deviation of the mean (n = 9). The description of glycan composition can be found in Figure 6.

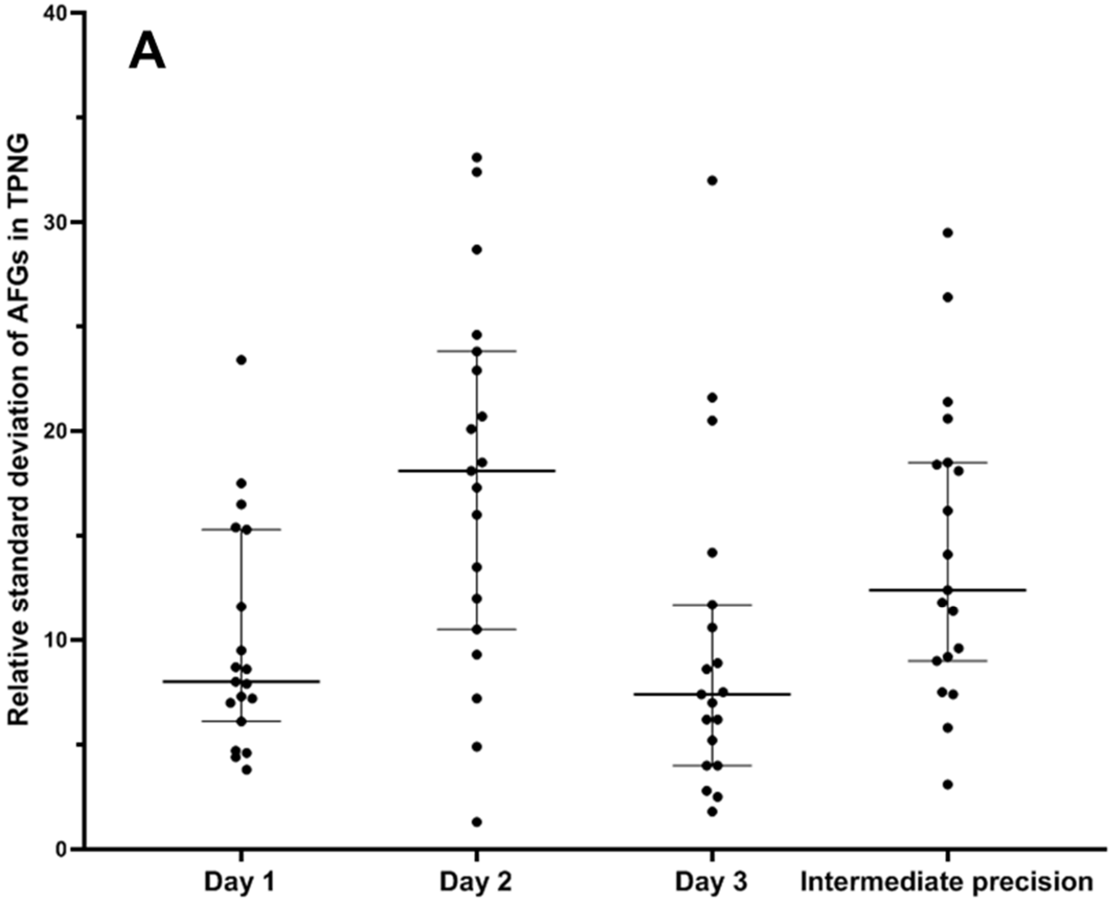

B

	Median	1st quartile	3rd quartile
Day 1; n = 9	8.0%	6.5%	13.4%
Day 2; n = 9	18.1%	11.3%	23.4%
Day 3; n = 9	7.4%	4.6%	11.1%
Intermediate precision; n = 3 x 9	12.4%	9.1%	18.5%

Supplementary Figure S27. Variation of the relative standard deviation (RSD) of the 19 antennary fucosylated glycans quantified in the interday repeatability study of the assay using MALDI-FT-ICR-MS for measurement. **(A)** The scatter dot plots shows the distribution of the RSD of the antennary fucosylated glycans on each day. Each point in a data set represents the RSD of an antennary fucosylated glycan for the sample replicates (n). The horizontal and vertical lines within each data set represents the median and 1st and 3rd quartiles, respectively. **(B)** The values for the median, 1st quartile and 3rd quartile are shown in the table.

A

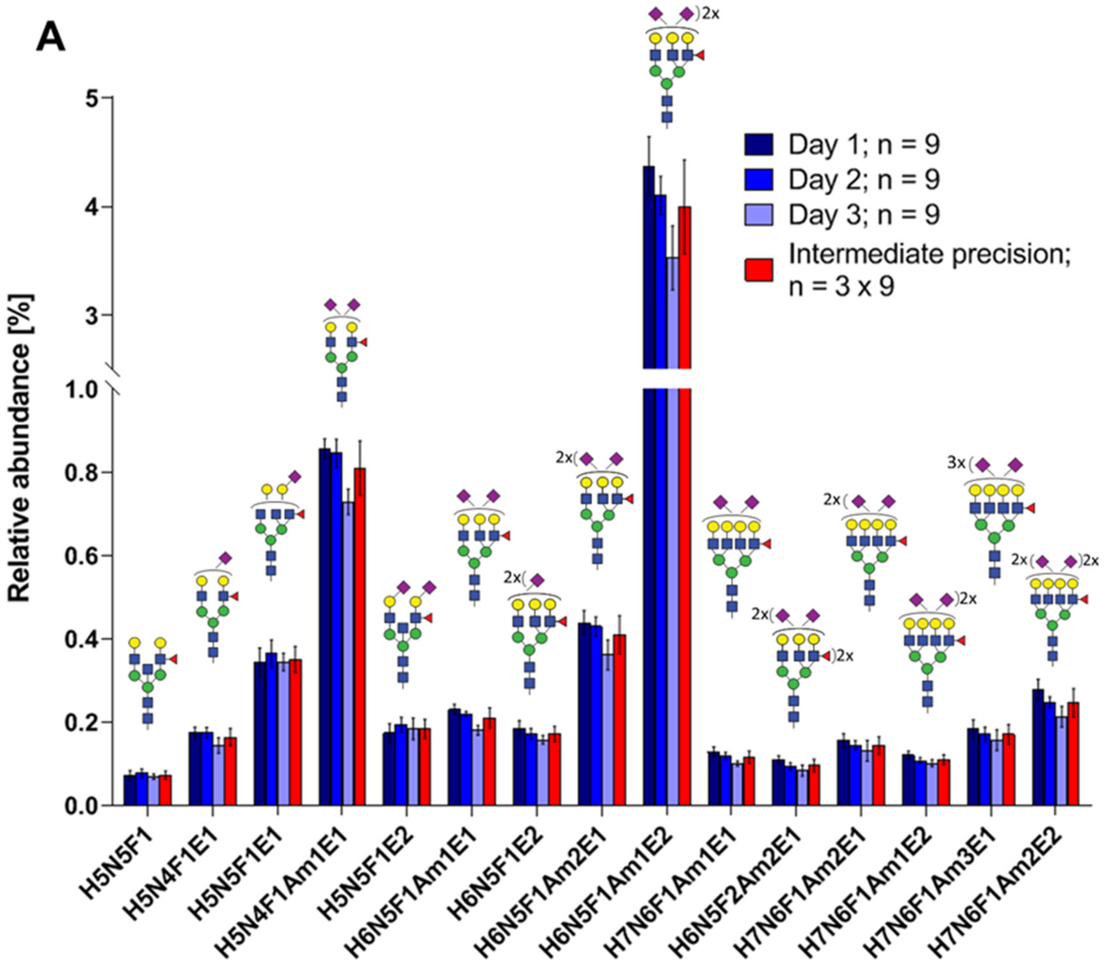

B

Relative standard deviations of the three most abundant AFGs in TPNG			
	H6N5F1Am1E2	H5N4F1Am1E1	H6N5F1Am2E1
Day 1; n = 9	6.3%	2.8%	6.6%
Day 2; n = 9	4.3%	4.0%	5.3%
Day 3; n = 9	8.3%	4.2%	10.0%
Intermediate precision; n = 3 x 9	10.8%	8.1%	11.0%

Supplementary Figure S28. Intermediate precision of sample preparation and MALDI-TOF-MS measurements of antennary fucosylated glycans in human TPNG. **(A)** The mean relative abundances of the 15 quantified antennary fucosylated glycans are shown with the error bars representing standard deviation (n = 9). **(B)** The relative standard deviations of the three most abundant antennary fucosylated glycans are shown. The descriptions of the glycan cartoons can be found in Figure 1. The description of glycan compositions can be found in Figure 6.

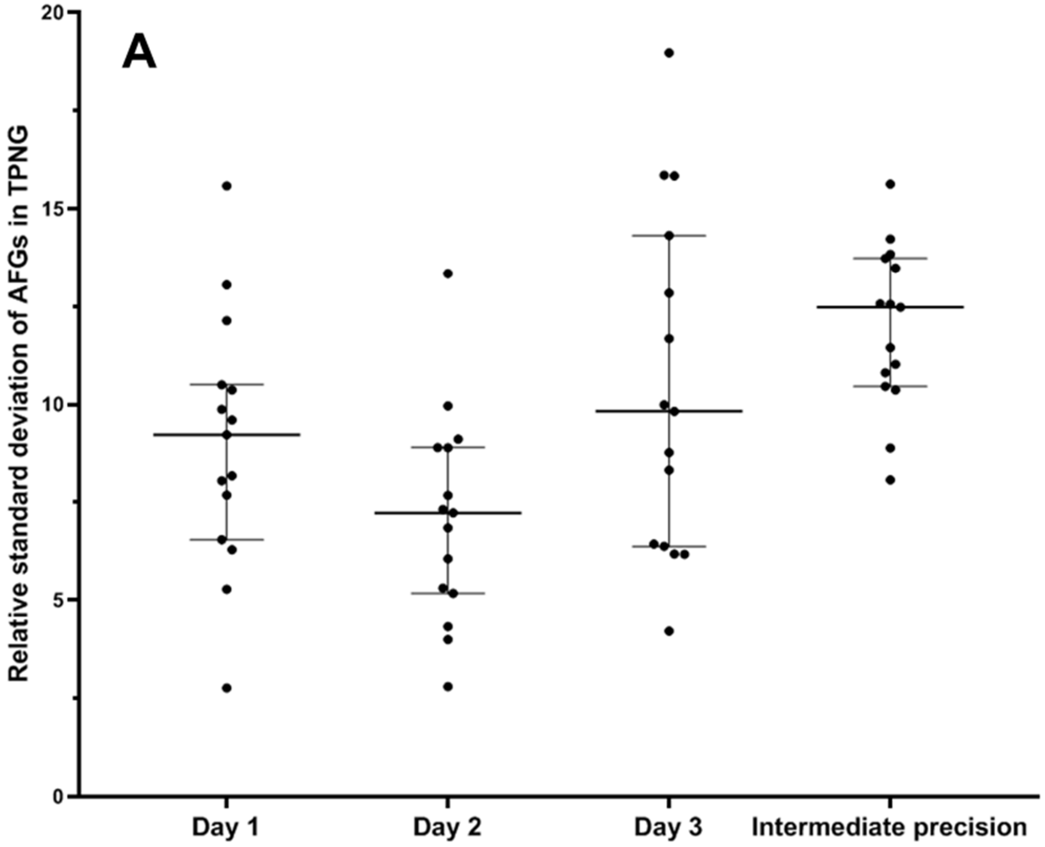

B

	Median	1st quartile	3rd quartile
Day 1; n = 9	9.2%	7.1%	10.4%
Day 2; n = 9	7.2%	5.2%	8.9%
Day 3; n = 9	9.8%	6.4%	13.6%
Intermediate precision; n = 3 x 9	12.5%	10.6%	13.6%

Supplementary Figure S29. Variation of the relative standard deviation (RSD) of the 15 antennary fucosylated glycans quantified in the interday repeatability study of the assay using MALDI-TOF-MS for measurement. **(A)** The scatter dot plots show the distribution of the RSD of the antennary fucosylated glycans on each day. Each point in a data set represents the RSD of an antennary fucosylated glycans for the sample replicates (n). The horizontal and vertical lines within each data set represents the median and 1st and 3rd quartiles, respectively. **(B)** The values for the median, 1st quartile and 3rd quartile are shown in the table.

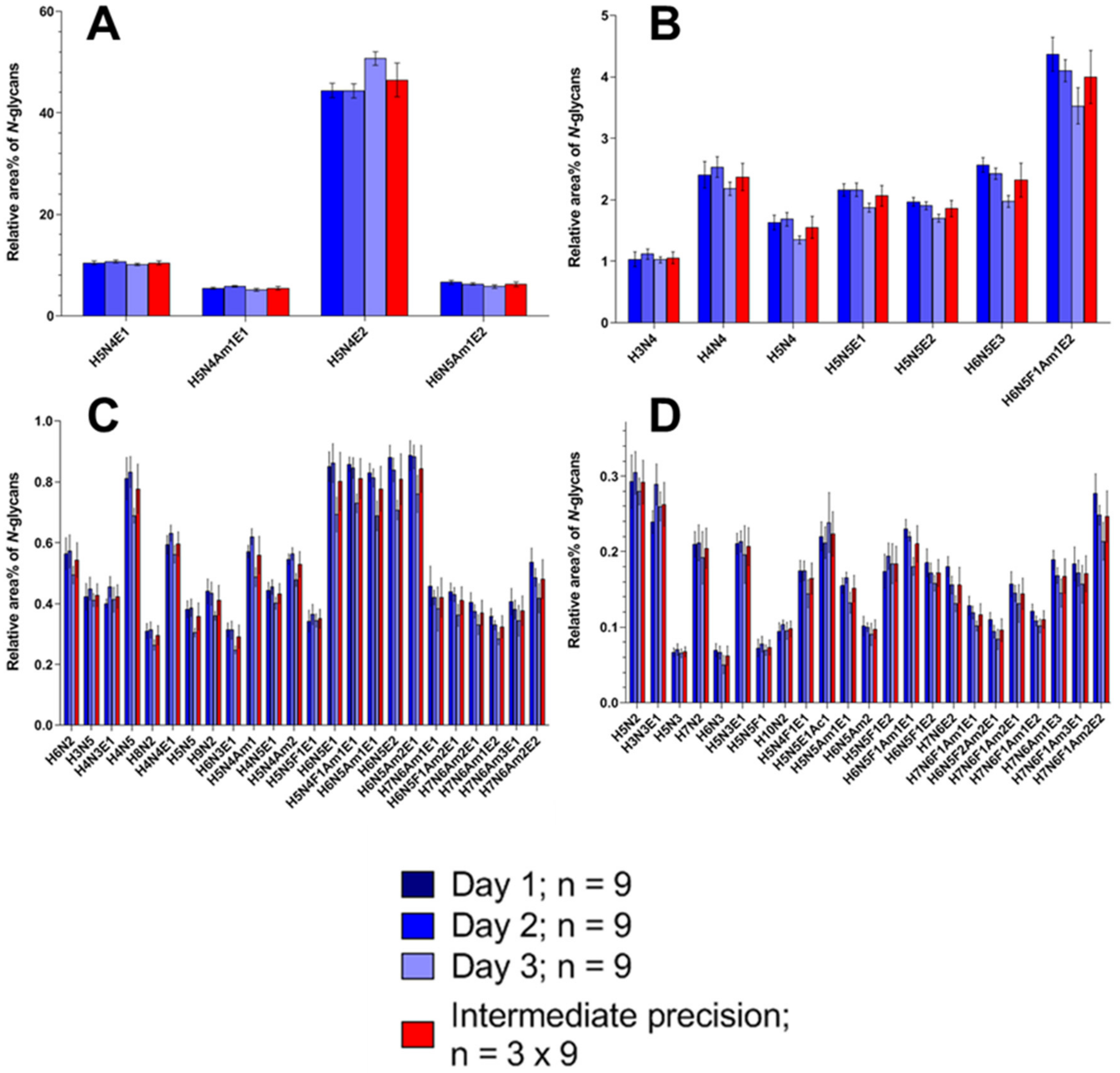

Supplementary Figure S30. Intermediate precision of sample preparation and MADLI-TOF-MS measurements for human TPNG using the antennary fucose assay. The 58 quantified glycans are sorted according to their relative area (%) **(A)** > 5%, **(B)** 5% to 1%, **(C)** 1% to 0.3%, **(D)** < 0.3%. The error bars show standard deviation of the mean (n = 9). The description of the glycan composition can be found in Figure 6.

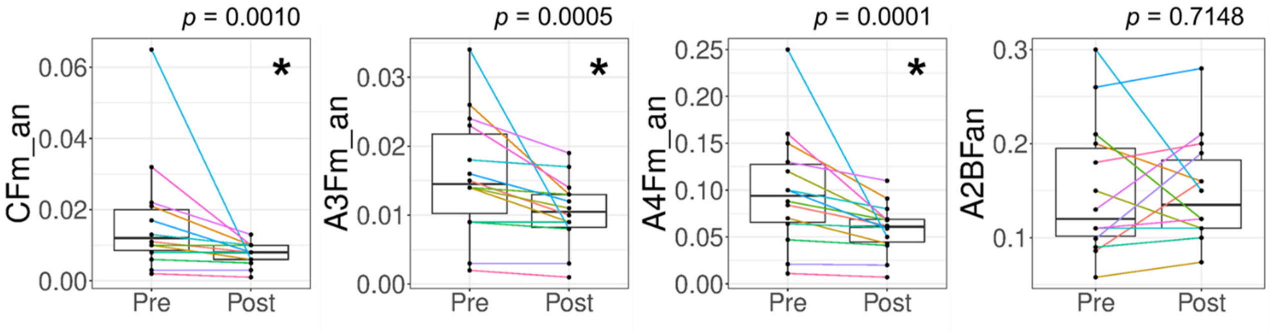

Supplementary Figure S31. Alterations in features of antennary fucosylated glycans for CRC patient samples. Derived traits for glycans were calculated to evaluate AF changes between the 14 pairs of pre-operative (Pre) vs. post-operative (Post) CRC patient samples. The patient samples were used in the antennary fucose assay and measurements made with MALDI-FT-ICR-MS. Significant changes were observed for multi-antennary fucosylation in complex *N*-glycans (**CFm_an**), multi-antennary fucosylation in triantennary glycans (**A3Fm_an**), multi-antennary fucosylation in tetraantennary glycans (**A4Fm_an**). No significant change was observed for antennary fucosylation in bisecting diantennary glycans (**A2BFan**). The *p* values shown are from a Wilcoxon matched-pairs signed-rank test with confidence level taken as 95%. Multiple-testing correction was performed using a false discovery rate (FDR) of 1% calculated with the Benjamini and Hochberg method. The *p* values < 0.0073 are considered significant, and are represented with an asterix (*).

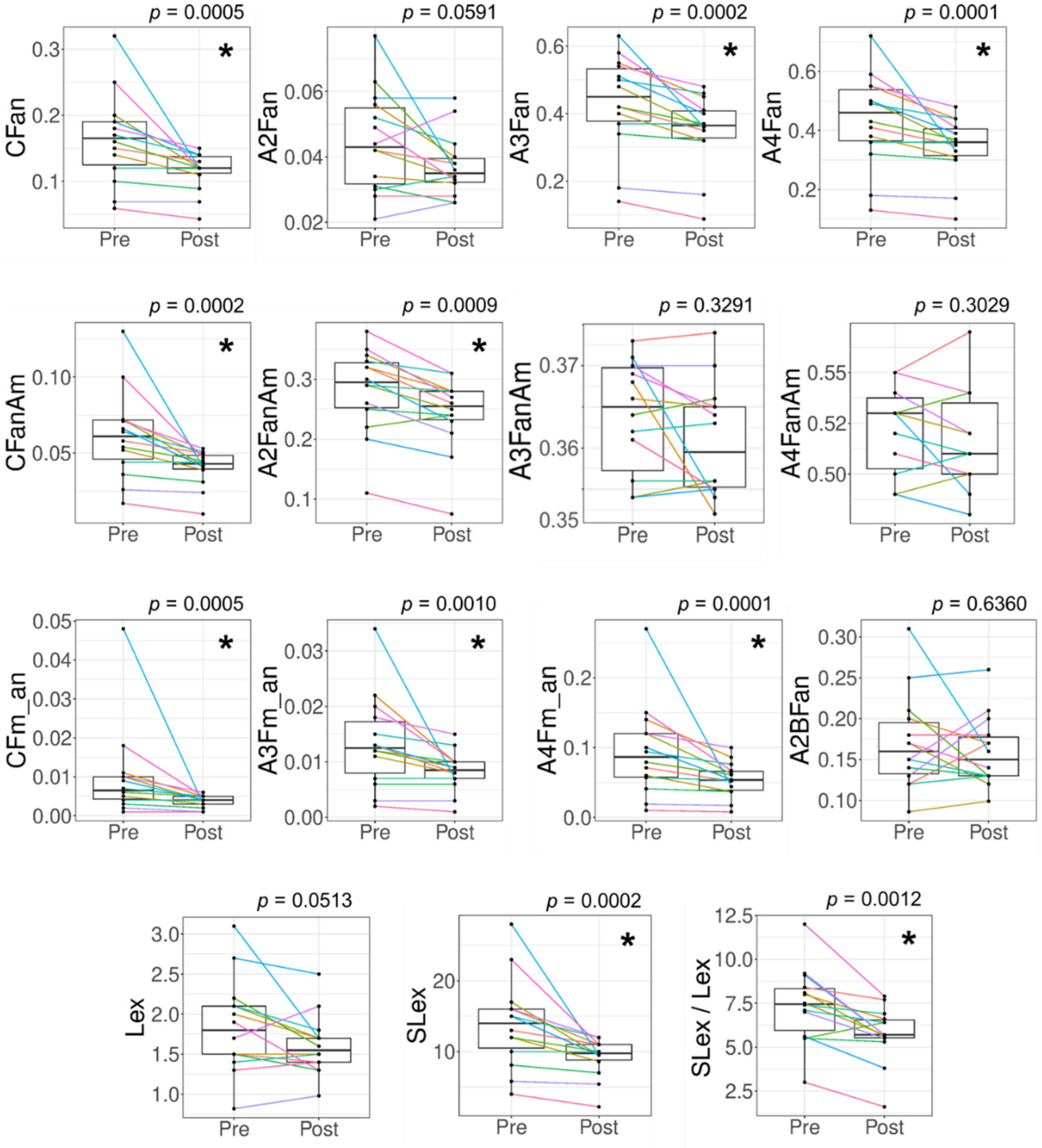

Supplementary Figure S32. Alteration in features of antennary fucosylated glycans for CRC patient samples. Derived traits for glycans were calculated to evaluate AF changes between the 14 pairs of pre-operative (Pre) vs. post-operative (Post) CRC patient samples. The patient samples were used in the antennary fucose assay and measurements made with MALDI-TOF-MS. Significant changes were observed for antennary fucosylation in complex *N*-glycans (**CFan**), multi-antennary fucosylation in complex *N*-glycans (**CFm_an**), antennary fucosylation in tri-antennary glycans (**A3Fan**), antennary fucosylation in tetra-antennary glycans (**A4Fan**), multi-AF in tri-antennary glycans (**A3Fm_an**), multi-antennary fucosylation in tetra-antennary glycans (**A4Fm_an**), α2,3-sialylation per antenna of total antennary fucosylated glycans (**CFanAm**), α2,3-sialylation per antenna of di-antennary antennary fucosylated glycans (**A2FanAm**), sialyl-Lewis x abundance

(**SLex**) in TPNG and the ratio of sialyl Lewis x to Lewis x abundances (**Slex / Lex**) in TPNG. No significant changes were observed for antennary fucosylation in di-antennary glycans (**A2Fan**), antennary fucosylation in bisecting di-antennary glycans (**A2BFan**), α2,3-sialylation per antenna of tri-antennary antennary fucosylated glycans (**A3FanAm**) and α2,3-sialylation per antenna of tetra-antennary antennary fucosylated glycans (**A4FanAm**) and Lewis x abundance (**Lex**) in TPNG. The *p* values shown are from a Wilcoxon matched-pairs signed-rank test with confidence level taken as 95%. Multiple-testing correction was performed using a false discovery rate of 1% calculated with the Benjamini and Hochberg method. The *p* values < 0.0067 are considered significant, and are represented with an asterix (*).

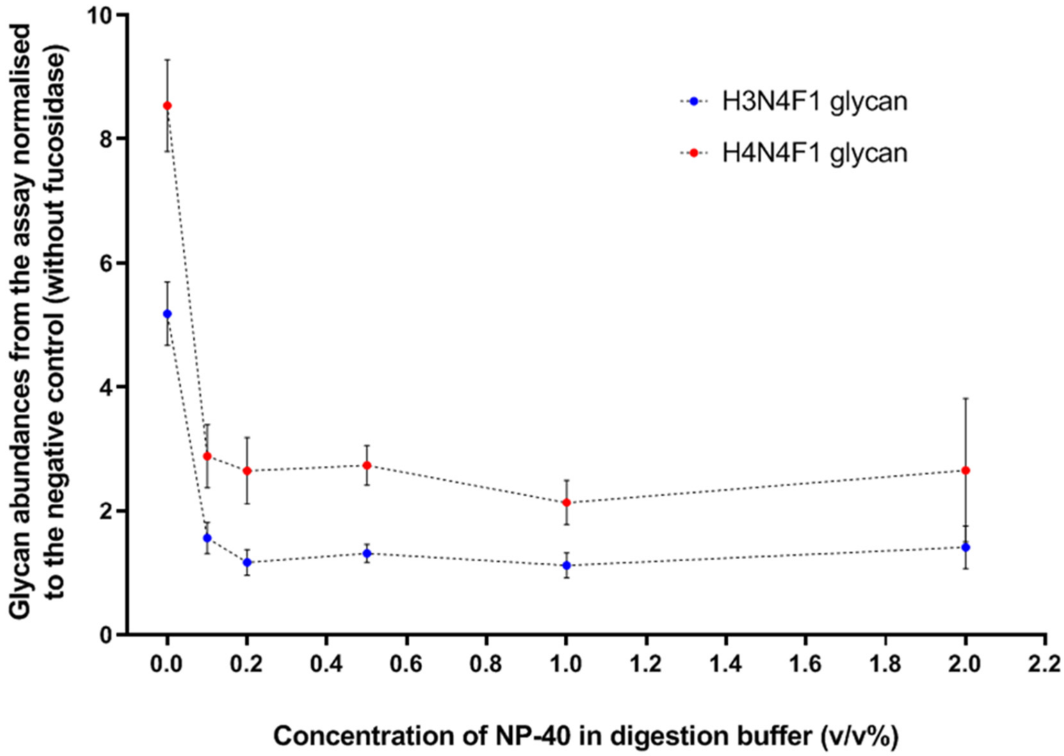

Supplementary Figure S33. Depletion of core fucosylation in TPNG by core fucosidase is improved with non-ionic detergent NP-40. The assay was performed without and with varying amounts of NP-40. MALDI-TOF-MS was used for the measurement of the assay. Core fucosylated glycans H3N4F1 and H4N4F1 were used to judge the depletion of core fucosylation using the formula: $\frac{Relative\ area\ (\%)\ of\ glycan\ after\ fucosidase\ treatment}{Relative\ area\ (\%)\ of\ glycan\ without\ fucosidase\ treatment}$. Each data point is the mean of three technical replicates and the error bars represents their corresponding standard deviation.

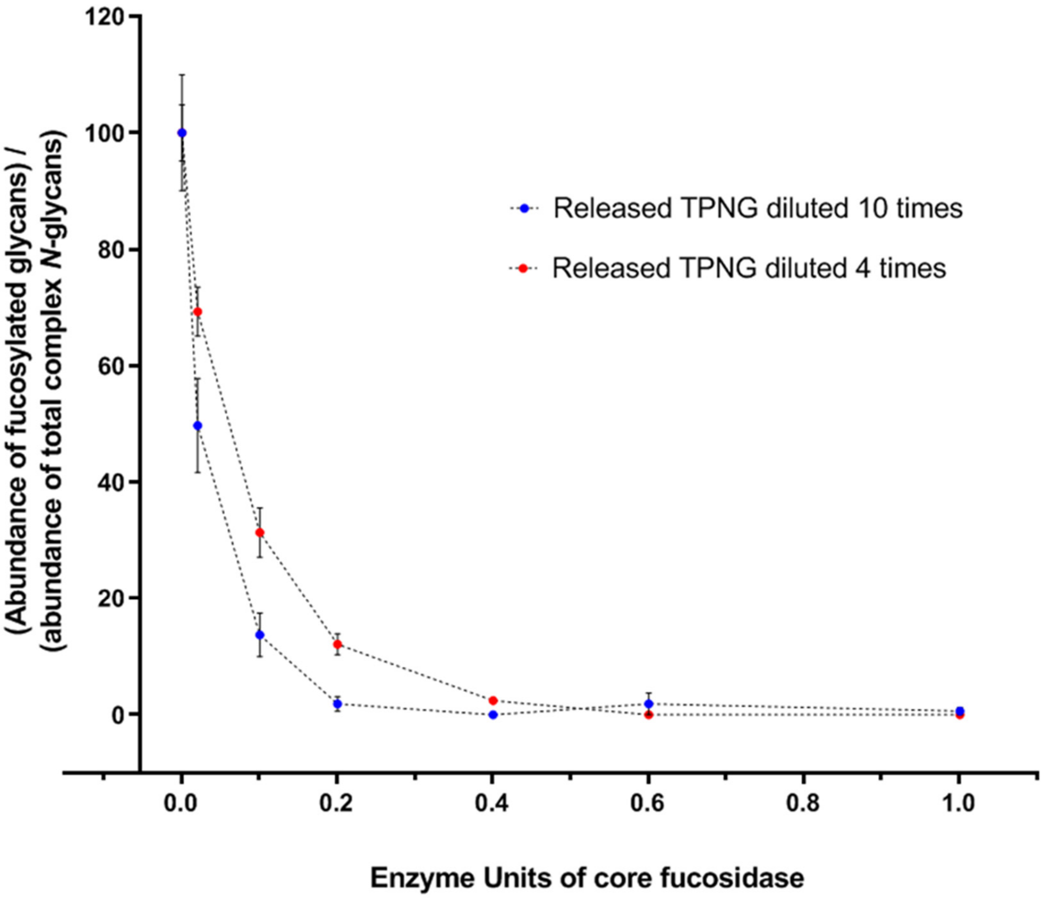

Supplementary Figure S34. Depletion of core fucosylation for varying dilutions of released human TPNG. Released TPNG were diluted 4 times [blue] and 10 times [red] in 1x acidic PBS before using it in the negative control (fucosidase untreated) or the assay with varying amounts of core fucosidase. MALDI-TOF-MS was used for measurements. Derived trait for total fucosylation in total complex *N*-glycans were calculated and the values were normalized within each data set. Each data point is the mean of three technical replicates and the error bars represents their corresponding standard deviation.

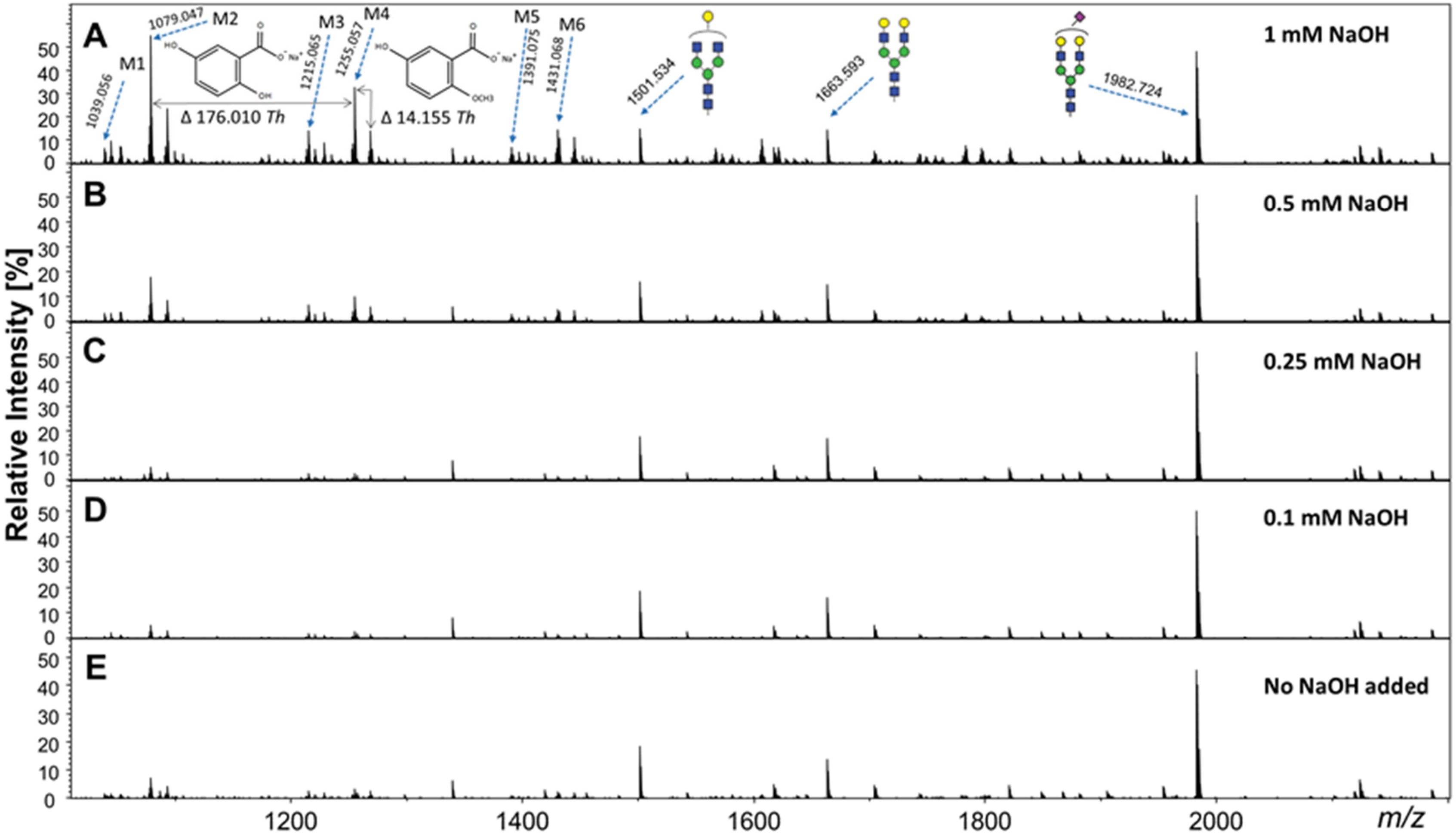

Supplementary Figure S35. Lowering of sodium concentration in MALDI matrix helps to reduce the abundance of matrix clusters in the MALDI-FT-ICR-MS spectra (*m/z* range 1000 - 2200) of the assay. Samples from the assay were spotted with super-DHB MALDI matrix containing either **(A)** 1 mM, **(B)** 0.5 mM, **(C)** 0.25 mM and **(D)** 0.1 mM NaOH or **(E)** no added NaOH. Abundant matrix clusters are annotated in the spectra by M1, M2, M3, M4, M5 and M6.

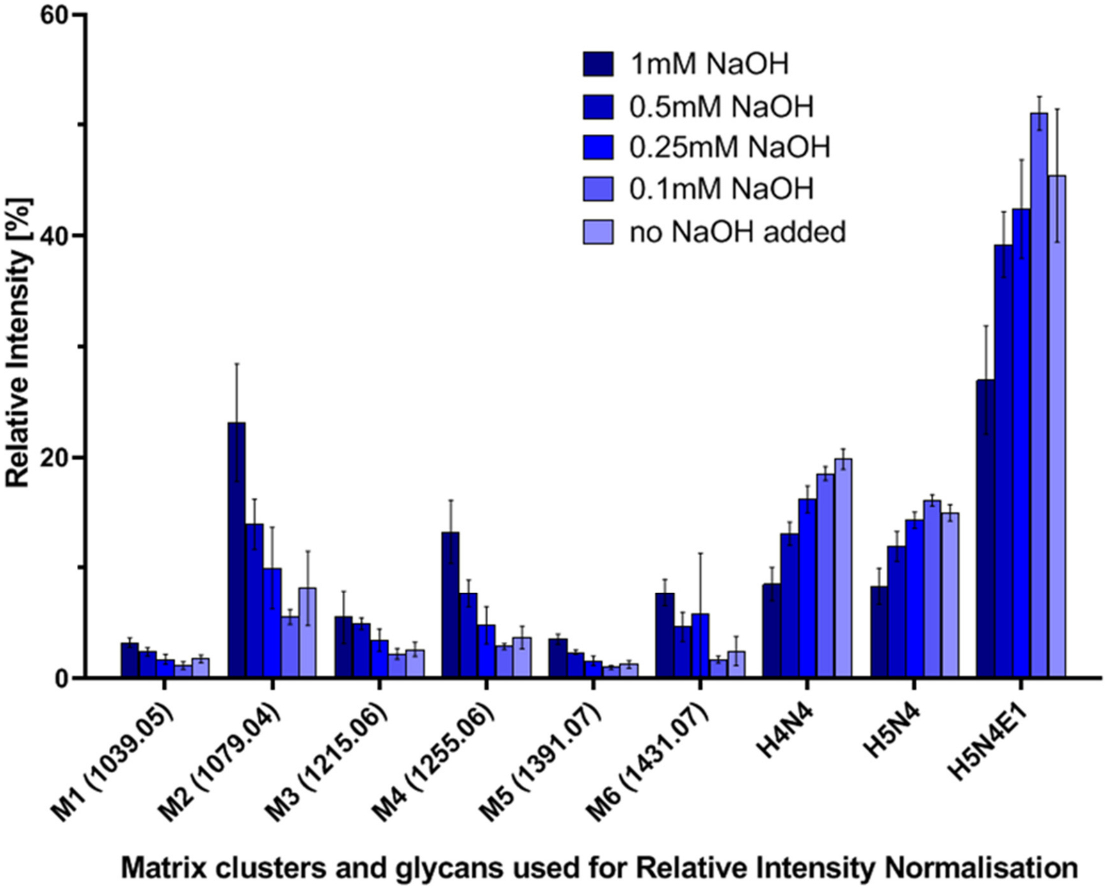

Supplementary Figure S36. Relative intensity (%) of matrix clusters in the MALDI-FT-ICR-MS spectra of the assay. Samples from the assay were spotted with the matrix solution of superDHB containing either 1 mM, 0.5 mM, 0.25 mM and 0.1 mM NaOH or no added NaOH. The relative intensity of the abundant matrix clusters M1, M2, M3, M4, M5 and M6 (annotated in Supplementary Figure S37) were normalized to the glycans H4N4, H5N4 and H5N4E1. The error bars represent the standard deviation of the mean (n = 6). The descriptions of glycan composition can be found in Figure 6.
